# Consequences of partially recessive deleterious genetic variation for the evolution of inversions suppressing recombination between sex chromosomes^1^

**DOI:** 10.1093/evolut/qpae060

**Published:** 2024-06-10

**Authors:** Colin Olito, Suvi Ponnikas, Bengt Hansson, Jessica K Abbott

**Affiliations:** Department of Biology, Lund University, Lund, Sweden; Department of Biology, Lund University, Lund, Sweden; Department of Biology, Lund University, Lund, Sweden; Department of Biology, Lund University, Lund, Sweden

**Keywords:** sex chromosomes, recombination, chromosomal inversion, mutation, indirect selection

## Abstract

The evolution of suppressed recombination between sex chromosomes is widely hypothesized to be driven by sexually antagonistic selection (SA), where tighter linkage between the sex-determining gene(s) and nearby SA loci is favored when it couples male-beneficial alleles to the proto-Y chromosome, and female-beneficial alleles to the proto-X. Although difficult to test empirically, the SA selection hypothesis overshadows several alternatives, including an incomplete but often-repeated “sheltering” hypothesis which suggests that expansion of the sex-linked region (SLR) reduces the homozygous expression of deleterious mutations at selected loci. Here, we use population genetic models to evaluate the consequences of partially recessive deleterious mutational variation for the evolution of otherwise neutral chromosomal inversions expanding the SLR on proto-Y chromosomes. Both autosomal and SLR-expanding inversions face a race against time: lightly-loaded inversions are initially beneficial, but eventually become deleterious as they accumulate new mutations, after which their chances of fixing become negligible. In contrast, initially unloaded inversions eventually become neutral as their deleterious load reaches the same equilibrium as non-inverted haplotypes. Despite the differences in inheritance and indirect selection, SLR-expanding inversions exhibit similar evolutionary dynamics to autosomal inversions over many biologically plausible parameter conditions. Differences emerge when the population average mutation load is quite high; in this case large autosomal inversions that are lucky enough to be mutation-free can rise to intermediate to high frequencies where selection in homozygotes becomes important (Y-linked inversions never appear as homozygous karyotypes); conditions requiring either high mutation rates, highly recessive deleterious mutations, weak selection, or a combination thereof.

## Introduction

Sex chromosomes have evolved from homologous pairs of autosomes repeatedly within many eukaryotic lineages across the tree of life (reviewed by Beukeboom & Perrin, 2014; Bachtrog et al., 2014). A striking feature of many sex chromosome systems is the evolution of recombination suppression, which profoundly influences the long-term fate of the chromosomes. Once recombination stops, the subsequent evolution of sequence divergence and functional degeneration of the non-recombining region of the sex-limited chromosome, and possibly dosage compensation to retain adequate gene expression levels in both sexes, can all contribute to the gradual evolution of sex chromosome heteromorphy (Bull, 1983; Rice, 1987, 1996; Bachtrog, 2006; Charlesworth et al., 2005a; Beukeboom & Perrin, 2014).

The initial loss of recombination between sex chromosomes is widely hypothesized to be caused by selection favoring linkage disequilibrium between the sex-determining gene(s) and nearby loci experiencing sex-differences in selection (e.g., sexually antagonistic loci with alleles that have opposite fitness effect between sexes) (Charlesworth & Charlesworth, 1980; Bull, 1983; Rice, 1987, 1996; Lenormand, 2003; Otto, 2019). Indeed, even though strong empirical support for the sexual antagonism hypothesis remains elusive, it continues to overshadow a variety of alternatives which have received less theoretical or empirical attention (Ponnikas et al., 2018; Olito & Abbott, 2023; Jefferies et al., 2021; Lenormand & Roze, 2022).

Several of these alternative hypotheses revolve around the idea that a chromosomal rearrangement––typically an inversion––expanding the male-limited region of a Y chromosome (or female-limited region of a W) may be selectively favored due to a form of heterozygote advantage, or “sheltering,” arising from the combination of wild-type and partially recessive deleterious alleles that it captures (Ironside, 2010; Ponnikas et al., 2018; Branco et al., 2017; Jay et al., 2022). In fact, a tangle of at least three distinct sheltering hypotheses have been described in varying detail, ranging from loose verbal models to mathematical and simulation models. First, Charlesworth and Wall (1999) cautiously suggested that selection might favor linkage between the sex-determining locus and multiple selected loci exhibiting pseudo-overdominance, an idea that was echoed in subsequent reviews (Ironside, 2010; Ponnikas et al., 2018). A later verbal model by Branco et al. (2017) proposed that partially recessive deleterious mutations in partial linkage with the sex-determining region could cause selection for recombination arrest to avoid homozygous expression in recombinant genotypes. More recently, Jay et al. (2022) modeled the evolution of inversions expanding the sex-linked region on Y chromosomes under deleterious mutation pressure using both deterministic models which assumed constant fitness effects for inversion genotypes depending on the alleles they initially capture, and accompanying individual-based simulations which did include recurrent mutations on inversions. Finally, in a new simulation model of regulatory degeneration and stabilization of inversions suppressing recombination between sex chromosomes, Lenormand and Roze (2022) modeled and verbally described the fixation of “lucky” (i.e., lightly loaded) inversions, and suggested that subsequent accumulation of new deleterious mutations due to selective interference would ultimately degrade their initial fitness advantage. Although Lenormand and Roze (2022) did not explicitly propose a “sheltering” mechanism, they also did not explain the selective mechanisms driving the fixation of “lucky” inversions beyond pointing out their initial fitness advantage (but see Lenormand & Roze, 2024, for some clarification). Critically, with the exception of Lenormand and Roze (2022), each of these earlier models proposes, in some way, that an inversion linking alleles at selected loci to the heterozygous male-determining allele on a Y chromosome will reduce the homozygous expression of partially recessive deleterious alleles at those loci (we review the various hypotheses in detail in Appendix A of the online supplementary material). This sheltering effect is hypothesized to cause higher fitness for inverted relative to non-inverted Y chromosomes.

The red thread running through each of these sheltering hypotheses is that deleterious mutational variation is pervasive throughout the genome (Muller, 1950; Crow, 1970; Charlesworth et al., 1993) and the fate of new inversions expanding the sex-linked region may therefore be strongly influenced by the random sample of that variation which they happen to capture. Moreover, deleterious genetic variation is known to have important implications for the evolution of inversions on autosomes: autosomal inversions undergo a complex time-dependent selection process that can result in diverse evolutionary outcomes, including fixation, extinction, and balanced polymorphism, depending on the set of alleles they initially capture (Nei et al., 1967; Charlesworth & Charlesworth, 1973; Connallon & Olito, 2022).

However, there is a crucial difference between autosomal inversions and those expanding the sex-linked region on a Y chromosome. By capturing the dominant sex-determining factor (or expanding the chromosomal region already linked to it), the latter are prevented from occurring in both X and Y chromosomes, and therefore always appear as heterozygous karyotypes in the male subpopulation. Several previous studies have suggested that this enforced heterozygosity of a proto-Y-linked inversion also applies to any partially recessive deleterious mutations initially captured by it, thereby paving the way for inversion fixation (Ironside, 2010; Jay et al., 2022). As we demonstrate below, this is an oversimplification. In fact, when partially recessive deleterious genetic variation is present, inversions expanding the sex-linked region (SLR hereafter) on Y chromosomes experience subtle differences in time-dependent selection processes compared to autosomal ones. Careful consideration of these time-inhomogeneous selection processes is necessary to fully understand the evolutionary dynamics of inversions contributing to recombination suppression between sex chromosomes.

In this article, we develop population genetic models to describe the evolutionary dynamics of new inversion mutations that capture the dominant sex-determining factor in randomly mating populations, while explicitly considering the consequences of standing deleterious mutational variation. We briefly describe the deterministic frequency dynamics predicted by the model, and compare these with autosomal inversions before turning our attention to the fixation probabilities for inversions of different lengths, which we calculate using stochastic Wright–Fisher (W–F) simulations. Our results illuminate two important features of inversions on Y chromosomes expanding the SLR: (i) because partially recessive deleterious mutations segregate outside the ancestral SLR on both X and Y chromosomes, mutations initially captured by an SLR-expanding inversion are expressed as homozygotes at the same frequency with which they occur in X chromosomes, so that inversions capturing even a single deleterious allele will carry a permanent deleterious mutation load; (ii) inversions initially capturing fewer than the average number of deleterious mutations over the chromosomal segment they span will initially be beneficial, but this selective advantage erodes over time as new mutations accumulate on descendant copies of the inversion. Eventually, this dwindling selective advantage becomes smaller than the permanent load carried by the inversion. At this point, the overall fitness effect of the inversion becomes irreversibly deleterious and its chances of fixation negligible. However, despite experiencing weaker counter-selection compared with autosomal inversions (due to the absence of homozygosity for inverted Y chromosomes), we find that in large finite populations (when N⁢s≫1), the fixation probability of SLR-expanding inversions capturing even a very small number of mutations approaches zero, resulting in essentially the same relation between inversion size and fixation probability as autosomal inversions for many biologically plausible parameter conditions. We close by discussing the implications of our findings for existing theories of recombination arrest between sex chromosomes.

## Methods and Results

### Overview of the model

Consider a population of diploid, randomly mating individuals with discrete generations, in which sex is determined genetically by a dominant male-determining factor (as in a male-heterogametic X–Y system). The model is equally applicable to female heterogametic Z–W systems if male/female labels are reversed. The order of life history events proceeds as follows: fertilization, mutation, selection, then meiosis. We model idealized proto sex chromosomes which can be divided into two regions: (i) a non-recombining SLR that could be limited to just the sex-determining gene(s) or a more extensive region harboring them; and (ii) a pseudoautosomal region (PAR) in which recombination can still occur, and functional homologs of any genes are still present on both X and Y chromosomes. Hence, our models are most applicable to genetic systems in which the evolution of recombination suppression between sex chromosomes is incomplete, and the PAR accounts for a sizeable fraction of the proto sex chromosomes (e.g., Charlesworth et al., 2005b).

We model the evolution of new chromosomal inversion mutations arising on a Y chromosome that would expand the SLR were they to fix among the Y chromosomes in the population. Our goal is to predict how new inversions will respond to indirect selection against deleterious mutations segregating within the population at the loci they span. To isolate these indirect selection effects, we assume that the inversion itself is neutral (i.e., inversions cause no breakpoint effects or meiotic dysfunction; Corbett-Detig 2016; Krimbas & Powell 1992; Olito & Abbott 2023; Villoutreix et al. 2021). For simplicity, we also assume that loci within the SLR do not contribute to indirect selection on inversions. This second assumption can be justified in different ways: (i) there has been sufficient differentiation and functional degeneration within the SLR on Y chromosomes that few functional genes remain in this region; (ii) the SLR is small relative to the length of inversions, such that there are few loci other than the sex-determining genes within the SLR; and (iii) any loci within the SLR that are captured by an inversion will contribute minimally to indirect selection favoring suppressed suppression because they are already fully sex-linked.

Our model relies on several other important simplifying assumptions. First, we assume that inversions completely suppress recombination between inverted-Y and X chromosomes over the chromosomal region they span (in fact, genetic exchange could occur via double crossovers or gene conversion; Krimbas & Powell 1992; Korunes & Noor 2019). Second, we assume that new inversions occur rarely enough that the evolutionary fate of a given inversion is independent of others (i.e., we assume “strong selection, weak mutation” with respect to inversions; Gillespie 1991). Our results therefore preclude the possibility that multiple inversions segregate simultaneously within the population. Third, we assume that deleterious alleles segregate at mutation–selection balance outside of the SLR, with no epistasis, and no linkage disequilibrium among selected loci or with the SLR prior to a new inversion mutation. This requires strong purifying selection against deleterious variants relative to mutation or genetic drift, and that deleterious mutations are not completely recessive or nearly so. Finally, we assume that fitness is multiplicative over the loci spanned by the inversion.

Below, we develop a deterministic model (i.e., for effectively infinite populations) describing the frequency dynamics of new inversions expanding the SLR on Y chromosomes in the presence of partially recessive deleterious mutational variation and illustrate important features of the model predictions. We emphasize the role of joint changes in the inversion frequency as well as deleterious allele frequencies on X chromosomes. For simplicity, we present deterministic results for the idealized case where mutation and selection coefficients at all loci spanned by the inversion are equal. We then present W–F simulations that incorporate stochastic fluctuations in inversion frequencies due to random gamete sampling in a finite population. We present most of the mathematical details in Appendices B–D of the online supplementary material. Computer code needed to reproduce the simulations and main figures is available on GitHub (https://github.com/colin-olito/shelteringOnSexChrom) and all versions of record are archived on Zenodo (Olito et al., 2024).

### Deterministic model

We first define two useful terms: the total number of selected loci located outside of the SLR on the chromosome arm containing it (i.e., within the PAR), $n_{\text{tot}}$, and the length of a new inversion, x, expressed as the proportion of the PAR that the new inversion links to the ancestral SLR. Assuming that the selected loci are distributed randomly along the chromosome arm, the number of loci spanned by a new inversion of length x will be $n=n_{\text{tot}}\,x$. Each of the n loci are assumed to be diallelic, with a wild-type allele, A, at the $i^{t\,h}$ locus that mutates to a deleterious variant, a, at a rate $\mu_{i}$ per meiosis (we ignore backmutation from a→A), with locus-specific genotypic relative fitnesses of $w_{i,A\,A}=1$, $w_{i,A\,a}=1-h_{i}\,s_{i}$, $w_{i,a\,a}=1-s_{i}$. Deleterious alleles segregate at each locus at their mutation–selection balance equilibrium frequency of ${\hat{q}}_{i}=\mu_{i}/\left(h_{i}\,s_{i}\right)$ (Haldane, 1927), and we focus our analysis on parameter conditions where the underlying assumptions of strong selection, weak mutation, and partial recessivity are well satisfied (i.e., where s≫μ and $h>\sqrt{\mu /s}$; Wright 1929). A new inversion mutation will capture a random sample of the standing deleterious variation at these n loci, which can be divided into two classes: loci where the inversion initially captures a deleterious allele (denoted by a superscript D) and those where it captures a wild-type allele (denoted by a superscript W).

To describe the deterministic frequency dynamics of an SLR-expanding inversion, it is necessary to track allele frequency changes for D and W loci within four different chromosome classes: X’s in ovules/eggs, X’s in pollen/sperm, non-inverted Y’s, and inverted Y’s, which we denote $X_{f}$, $X_{m}$, Y, and $Y^{I}$, respectively (see Otto, 2014; Olito & Abbott, 2023). The frequencies at time t for each of the n loci can be described using the following notation: $q_{X_{f},t}^{D,i}$, $q_{X_{m},t}^{D,i}$, $q_{Y,t}^{D,i}$, $q_{Y^{I},t}^{D,i}$, and $q_{X_{f},t}^{W,i}$, $q_{X_{m},t}^{W,i}$, $q_{Y,t}^{W,i}$, $q_{Y^{I},t}^{W,i}$, where q refers to the deleterious allele frequency at the i⁢th locus. Note that, by assumption, $q_{Y^{I}}^{D,i}=1$ for all t because all descendant copies of the inversion will carry deleterious alleles at D loci. Likewise, $q_{Y^{I}}^{W,i}=0$ for t=1 but increases due to new mutations on descendant copies of the inversion. Note that recombination between X chromosomes in eggs/ovules does not alter haplotype frequencies in this model (Otto, 2014; Otto et al., 2011). We present the full development of the recursions in Appendix B of the online supplementary material.

Under the simplifying assumption that mutation and selection parameters are constant across all loci captured by the inversion ($\mu_{i}=\mu$, $s_{i}=s$, $h_{i}=h$), the number of deleterious alleles initially captured by the inversion, d, will be Poisson distributed: d∼Poisson⁢(U⁢x/(h⁢s)), where $U=\mu \,n_{\text{tot}}$ is the chromosome-arm wide mutation rate and U⁢x is the mutation rate over the region spanned by the inversion (Haldane, 1937; Kimura & Maruyama, 1966). In this idealized case, the deleterious allele frequencies at all loci within a given chromosomal class will follow the same trajectory (e.g., each inversion-captured locus that is initially free of a deleterious allele will evolve the same as other loci in the inversion with the same, mutation-free initial state). That is, $q_{\text{class},t}^{W,i}=q_{\text{class},t}^{W}$, and $q_{\text{class},t}^{D,i}=q_{\text{class},t}^{D}$, where $\text{class}\in \left\{X_{f},X_{m},Y,Y^{I}\right\}$. This allows us to define the following simplified recursion for the frequency of an inversion that initially captures d deleterious alleles in terms of the allele frequencies:


$$\begin{array}{cc} Y_{t+1}^{I} & =Y_{t}^{I}[{\left(1-s\left(hp_{X_{f,t}}^{D}+q_{X_{f,t}}^{D}\right)\right)}^{d}\\ & {\left(1-s\left(h\left(p_{Y_{t}^{I}}^{W}q_{X_{f,t}}^{W}+q_{Y_{t}^{I}}^{W}p_{X_{f,t}}^{W}\right)+q_{Y_{t}^{I}}^{W}q_{X_{f,t}}^{W}\right)\right)}^{n-d}]/\bar{w}{}^{Y} \end{array}$$ (1)

where


$$\begin{array}{cc} {\bar{w}}^{Y} & =Y_{t}^{I}[{\left(1-s\left(hp_{X_{f,t}}^{D}+q_{X_{f,t}}^{D}\right)\right)}^{d}\\ & {\left(1-s\left(h\left(p_{Y_{t}^{I}}^{W}q_{X_{f,t}}^{W}+q_{Y_{t}^{I}}^{W}p_{X_{f,t}}^{W}\right)+q_{Y_{t}^{I}}^{W}q_{X_{f,t}}^{W}\right)\right)}^{n-d}]\\ & +\left(1-Y_{t}^{I}\right)[{\left(1-s\left(h\left(p_{X_{f,t}}^{D}q_{Y,t}^{D}+q_{X_{f,t}}^{D}p_{Y,t}^{D}\right)+q_{X_{f,t}}^{D}q_{Y,t}^{D}\right)\right)}^{d}\\ & {\left(1-s\left(h\left(p_{X_{f,t}}^{W}q_{Y,t}^{W}+q_{X_{f,t}}^{W}p_{Y,t}^{W}\right)+q_{X_{f,t}}^{W}q_{Y,t}^{W}\right)\right)}^{n-d}], \end{array}$$ (2)

and we have used the convention $p_{\cdot ,t}^{\cdot }=1-q_{\cdot ,t}^{\cdot }$ to simplify notation. The deterministic frequency dynamics of the inversion can now be fully described by a system of eight recursions corresponding to the deleterious allele frequencies in each of the seven relevant loci × chromosome classes ($q_{X_{f}}^{\prime ,D}$, $q_{X_{m}}^{\prime ,D}$, $q_{Y}^{\prime ,D}$, and $q_{X_{f}}^{\prime ,W}$, $q_{X_{m}}^{\prime ,W}$, $q_{Y}^{\prime ,W}$, $q_{Y^{I}}^{\prime ,W}$; see Appendix B of the online supplementary material) and the frequency of the inversion, *Equations 1* and 2.

*Equation 1* offers immediate insight into how D vs. W loci contribute to fitnesses of inverted relative to non-inverted haplotypes. The fitness effects of D loci depend solely on the frequency of deleterious alleles in X chromosomes in ovules/eggs (selection terms in bracketed expressions with an exponent of d involve only $p_{X_{f},t}^{D}$ and $q_{X_{f},t}^{D}$) because all descendant copies of the inversion already carry a deleterious allele at these loci. In an infinite population under recurrent mutation with no back-mutation (as we have assumed throughout), $q_{X_{f},t}^{D}$ will always be non-zero, and so it is immediately clear that D loci will impart a permanent fitness cost to the inversion. Meanwhile, W loci depend jointly on the frequency of deleterious alleles in ovule/egg-derived X chromosomes and the accumulation of new deleterious mutations on descendant copies of the inversion at these loci (selection terms involve $p_{X_{f},t}^{W}$, $q_{X_{f},t}^{W}$, and $q_{Y^{I},t}^{W}$). Compared to the fitness of standard Y chromosomes (the second expression in square brackets in *Equation 2*), it is clear that any temporary fitness advantage of new inversions must come from the initially low frequency of deleterious alleles at W loci among inverted Y chromosomes ($q_{Y^{I},t}^{W}$). The key questions become: when, and for how long, does the temporary fitness benefit from W loci outweigh the permanent load associated with D loci? and does it result in elevated fixation probabilities for new SLR-expanding inversions relative to autosomal ones?

### Autosomal inversions

To make a fair comparison with the behaviour of autosomal inversions, it is necessary to take the frequency changes of deleterious alleles in both inverted and non-inverted chromosomes into account, as we have done for SLR-expanding inversions above. Nei et al. (1967) developed a full set of exact recursions for initially mutation-free autosomal inversions which takes into account all of the relevant gene frequencies for the special case of completely recessive deleterious mutations. In the supplementary material (Appendix C), we extend the model of Nei et al. (1967) to accommodate partially recessive deleterious mutations, and the possibility that deleterious mutations are captured at d of the n loci spanned by the inversion. The resulting exact recursion for the per-generation frequency change of an autosomal inversion is


$$\begin{array}{cc} I_{t+1}=[ & I_{t}^{2}\,{\left(1-s\right)}^{d}\,{\left(1-s\,\left(2\,h\,Q_{I}^{W}\,P_{I}^{W}+Q_{I}^{W\,2}\right)\right)}^{n-d}\\ & +I_{t}\,\left(1-I_{t}\right)\,{\left(1-s\,\left(h\,P_{N}^{D}+Q_{N}^{D}\right)\right)}^{d}\\ & {\left(1-s\left(h\left(Q_{N}^{W}P_{I}^{W}+P_{N}^{W}Q_{I}^{W}\right)+Q_{N}^{W}Q_{I}^{W}\right)\right)}^{n-d}]/\bar{w}{}_{I}, \end{array}$$ (3)

where ${\bar{w}}_{I}$ is equal to


$$\begin{array}{cc} {\bar{w}}_{I}= & I_{t}^{2}\,\left[{\left(1-s\right)}^{d}\,{\left(1-s\,\left(2\,h\,Q_{I}^{W}\,P_{I}^{W}+Q_{I}^{W\,2}\right)\right)}^{n-d}\right]\\ & +2I_{t}\left(1-I_{t}\right)[{\left(1-s\left(2hP_{N}^{D}+Q_{N}^{D}\right)\right)}^{d}\\ & {\left(1-s\left(h\left(Q_{N}^{W}P_{I}^{W}+P_{N}^{W}Q_{I}^{W}\right)+Q_{N}^{W}Q_{I}^{W}\right)\right)}^{n-d}]\\ & +{\left(1-I_{t}\right)}^{2}[{\left(1-s\left(2hQ_{N}^{D}P_{N}^{D}+Q_{N}^{D\,2}\right)\right)}^{d}\\ & {\left(1-s\left(2hQ_{N}^{W}P_{N}^{W}+Q_{N}^{W\,2}\right)\right)}^{n-d}], \end{array}$$ (4)

where I denotes the inversion frequency, $Q_{\cdot }^{\cdot }$ denotes the relative frequency of deleterious alleles at each selected locus on inverted or non-inverted chromosomes (denoted with subscript I or N), and we retain the superscript W and D notation to indicate loci at which a wild-type or deleterious alleles is initially captured by the inversion.

Comparison of the recursions for SLR-expanding (*Equation 1*) and autosomal inversions (*Equation 3*) suggests when SLR-expanding inversions will behave differently than autosomal ones. Specifically, the recursion for SLR-expanding inversions is essentially identical to the terms involving selection in autosomal inversion heterozygotes in *Equation 3*. Hence, a reasonable expectation would be that SLR-expanding and autosomal inversions will behave very similarly except when inversions deterministically rise to high enough frequencies that selection in autosomal inversion homozygotes becomes important; conditions that require very weak selection, highly recessive deleterious mutations, or both (i.e., h⁢s must be very small).

### Deterministic frequency dynamics

When deleterious alleles are approximately codominant (i.e., $h_{i}\approx 1/2$), most purifying selection occurs in heterozygotes and an inversion initially loaded with even a single deleterious allele (i.e., when d>0) will not invade (Connallon & Olito 2022, see, Appendix B). However, when deleterious mutations are partially recessive ($0<h_{i}<1/2$), as expected by theory and supported by empirical data (Manna et al. 2011; Agrawal & Whitlock 2011; Huber et al. 2018; reviewed in Billiard et al. 2021), selection in homozygotes becomes more important, and both lightly loaded autosomal and SLR-expanding inversions on Y chromosomes can deterministically rise to high frequency under certain conditions.

Two analytic results for the special case of initially mutation-free inversions (where d=0) under mutation–selection balance provide useful benchmarks against which to compare our results: (i) the initial relative fitness of a mutation-free inversion will be approximately 1+U⁢x; and (ii) the long-term deterministic frequency of initially unloaded inversions after their initial fitness benefit has eroded (the effective initial frequency *sensu* Connallon & Olito 2022) will be approximately equal to $q^{*}\approx q_{0}\,e^{U\,x/\left(h\,s\right)}+O\,\left(N^{-2}\right)$, where $q_{0}$ is the initial frequency of the inversion (i.e., $q_{0}=2/N$ for a single-copy inversion mutation on a Y chromosome; see Appendix B.4 of the online supplementary material for derivations of these approximations).

**Figure 1:**
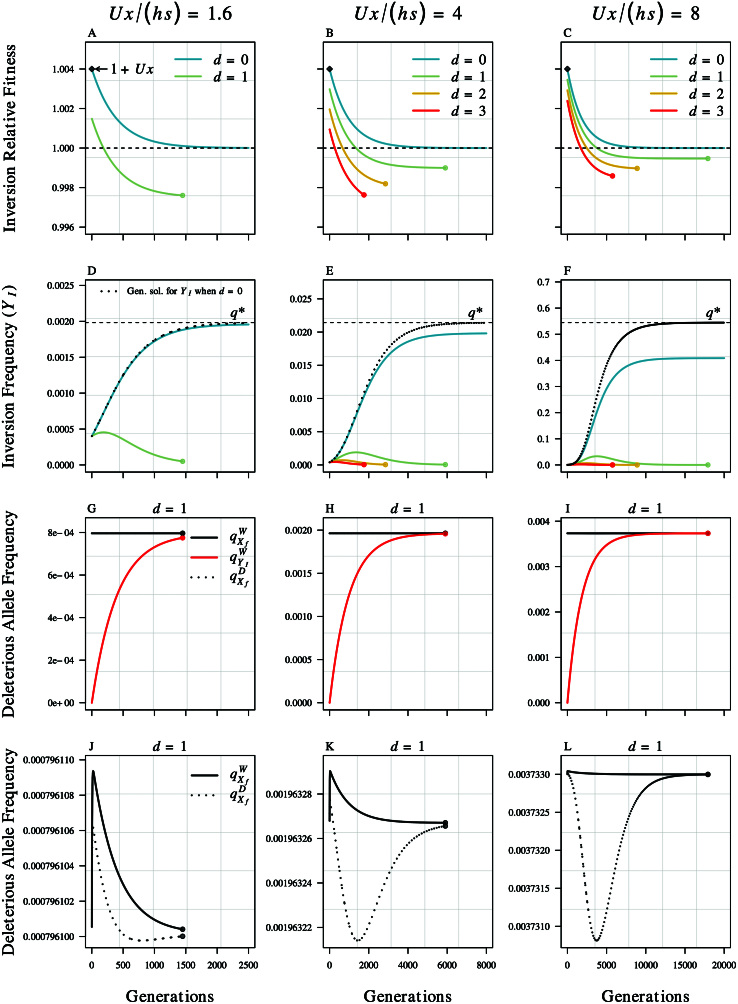
Illustration of deterministic fitness and frequency dynamics for inversions initially capturing different numbers of deleterious alleles (i.e., different d values). Results are shown for inversions of length x=0.2, and three different dominance scenarios (h = {0.25, 0.1, 0.05}, yielding average deleterious mutation loads over the chromosomal segment spanned by inversions of U⁢x/(h⁢s)= {1.6, 4, 8}, corresponding to each column of panels, left to right). (A–C) Fitness of SLR-expanding inversions on a Y chromosome relative to the average fitness of all Y chromosomes (color-coded lines). Colored points indicate when the corresponding inversions dropped below a frequency of ${\text{𝟣𝟢}}^{-\text{𝟧}}$, where they were considered to be extinct. Black diamonds indicate the analytic approximation for the relative fitness of unloaded inversions (d=𝟢) in the first generation, and the horizontal dashed line benchmarks a relative fitness of one. (D–F) Inversion frequency dynamics (color-coded lines) and illustrate that despite being initially beneficial, lightly loaded inversions (*i*) are not expected to deterministically rise to high frequencies unless deleterious mutations are strongly recessive, selection is very weak, or both (i.e., h⁢s is small, right-hand column); and (*ii*) will eventually become deleterious and crash to extinction (see also Figures S1–S3 in Appendix E of the online supplementary material). Horizontal dashed lines benchmark the asymptotic frequency of initially unloaded inversions, ${\text{𝑞}}^{*}$, while dotted lines show the corresponding discrete-time general solution for $Y_{I,t}$ from which ${\text{𝑞}}^{*}$ is derived. (G–I) Deleterious allele frequency dynamics at W loci on the inversion ($q_{Y_{I}}^{W}$; red line), and both W and D loci on X chromosomes in ovules/eggs ($q_{X_{f}}^{W}$ and $q_{X_{f}}^{D}$; black solid and dashed lines, respectively) for the representative case of inversions initially loaded with a single deleterious allele. (J–L) Visualize the perturbations to deleterious allele frequencies on X chromosomes in ovules, which are very small relative to the equilibrium frequency, and therefore not visible in (G–I). Results were generated using the following parameter values: s=0.01, U=0.02, x=0.2, and $n_{t\,o\,t}={\text{𝟣𝟢}}^{\text{𝟦}}$.

Figure 1 illustrates key features of the deterministic dynamics for initially beneficial SLR-expanding inversions capturing different numbers of partially recessive deleterious mutations for three dominance scenarios ($h_{i}=h=\left\{0.25,0.1,0.05\right\}$, corresponding to average mutation loads over the chromosomal segment spanned by inversions of U⁢x/(h⁢s)={1.6,4,8}). The time course of inversion fitness relative to non-inverted Y chromosomes (Figure 1A–C) illustrates both the decay of the initial fitness benefit as new mutations accumulate on descendant copies of the inversion-bearing chromosome at W loci (inversions that initially have relative fitness greater than one) and the permanent deleterious load due to D loci (all inversions with d>0 eventually become deleterious). In the best case scenario, initially mutation-free inversions start with a relative fitness of approximately 1+U⁢x, which degrades until they ultimately become neutral. The tipping-point where the relative fitness of loaded inversions drops below one occurs when the transient benefit to the inversion of initially capturing fewer than the average number of deleterious mutations no longer compensates for the cost of being fixed for those few mutations. The deterministic inversion frequency dynamics (Figure 1D–F) reflect these changes in relative fitness and highlight that initially unloaded inversions asymptotically converge on $q^{*}$ when h⁢s is large, and also show that the analytic approximations for $Y_{I}$ and $q^{*}$ increasingly overestimate the asymptotic frequency of initially unloaded inversions when deleterious mutations are more strongly recessive. Nevertheless, when deleterious mutations are more strongly recessive (e.g., h=0.05), and the average number of deleterious mutations carried by a chromosome is therefore quite high, inversions capturing several deleterious mutations can deterministically rise to intermediate frequencies before the return to equilibrium deleterious allele frequencies on inverted haplotypes causes them to become deleterious and crash to extinction (Figure 1C, F, and I).

The deleterious load carried by inversions due to D loci is temporally dynamic because the frequencies of partially recessive deleterious alleles on X (and non-inverted Y) chromosomes change over time in response to the inversion frequency (Figure 1G–I). We illustrate this for representative cases of inversions initially loaded with relatively few deleterious mutations (d=1 in Figure 1G–I) by showing the deterministic frequency dynamics for three important loci × chromosome classes: W loci on the inversion ($q_{Y_{I}}^{W}$), and both W and D loci on X chromosomes in ovules/eggs ($q_{X_{f}}^{W}$ and $q_{X_{f}}^{D}$). The red line shows the accumulation of deleterious mutations on the inversion at W loci, which causes the decline in initial fitness benefit due to these loci. The black solid and dashed lines show the corresponding changes in deleterious allele frequency among ovule/egg-derived X chromosomes at W loci ($q_{X_{f}}^{W}$) and D loci ($q_{X_{f}}^{D}$), respectively. Since $q_{Y^{I}}^{W}$ is initially 0, the inversion rises in frequency, and as it does the total frequency of deleterious alleles drops below the equilibrium value of μ/(h⁢s). The resulting imbalance causes the frequency of deleterious alleles on X chromosomes ($q_{X_{f}}^{W}$ and $q_{X_{f}}^{D}$) to be temporarily perturbed away from their initial equilibrium value (Figure 1J–L). At W loci on X chromosomes, $q_{X_{f}}^{W}$ temporarily increases as $q_{Y^{I}}^{W}$ returns equilibrium, although the effect is quite small at each locus. In contrast, selection against deleterious alleles at D loci on ovule/egg-derived X chromosomes intensifies as the initially beneficial inversion increases in frequency because all inverted-Y-bearing sons who inherit a deleterious allele from their mother’s X chromosome will be homozygous for the deleterious allele at D loci. This intensified selection drives the frequency of deleterious alleles at D loci on X chromosomes ($q_{X_{f}}^{D}$) down to lower levels relative to the equilibrium prior to the inversion. Nevertheless, the deleterious load due to D loci persists because new mutations continually arise on X and non-inverted Y chromosomes. When the transient benefit of the inversion due to W loci can no longer compensate for this load, the inversion becomes deleterious and declines in frequency, at which point the deleterious allele frequencies return to the pre-inversion equilibrium (see also Figures S4–S9 in Appendix E of the online supplementary material).

The deterministic dynamics of autosomal inversions confirm the intuition from *Equation 1* and *Equation 3* that they will behave similarly to SLR-expanding inversions unless h⁢s is very small, with the differences exacerbated for larger inversions. Interestingly, the effects of the inversion dynamics on the frequencies of deleterious alleles on non-inverted chromosomes are stronger for autosomal inversions than for SLR-expanding inversions. The reason is that, for autosomal inversions, all non-inverted chromosomes have a non-zero chance of pairing with a segregating inverted chromosome in each generation, while, for SLR-expanding inversions, only 1/3 of all X chromosomes (those carried by males) have a possibility of pairing with an inverted Y. An overview of the deterministic dynamics for both autosomal and SLR-expanding inversions of different lengths and initially loaded with different numbers of deleterious alleles is presented in Appendix E, Figures S1–S15, of the online supplementary material.

### W–F simulations

While the deterministic frequency dynamics presented above clearly illustrate the shifting balance between the time-dependent selection processes on inversions due to W and D loci, they do not consider either the stochastic process of gamete sampling present in finite populations which can result in loss of rare inversions, nor the likelihood that a new inversion captures a given number of mutations. In reality, larger inversions are more likely to capture a greater number of deleterious mutations (see Figures S4–S15 of the online supplementary material), and the combined effects of W and D loci on inversion relative fitness will depend on allele frequency dynamics at each of the n loci.

To begin tackling the effects of drift, we use W–F simulations carried out in R (R Core Team, 2023) to estimate the fixation probability for a single Y chromosome bearing an SLR-expanding inversion as a function of inversion length. We follow the simulation approach of Connallon and Olito (2022), which relies upon several important simplifying assumptions. As in our deterministic models, our W–F simulations focus on the idealized case where the fitness effects of deleterious mutations are constant across loci and combine multiplicatively; an approach that is widely used in multilocus models of mutation–selection balance (e.g., Haldane, 1937; Nei et al., 1967; Bachtrog, 2008). Due to the computation time necessary to perform the large number of replicate simulations required to accurately estimate small fixation probabilities, our simulations do not track individual deleterious mutations at selected loci. Instead, we incorporate the effects of deleterious mutations on inversion dynamics by modelling temporal changes in selection coefficients for inversions under mutation–selection balance. Specifically, we assume that deleterious mutations on non-inverted Y and all X chromosomes remain at equilibrium frequencies ($\hat{q}=\mu /\left(h\,s\right)$), while deleterious alleles at W loci among inverted haplotypes deterministically return to equilibrium during the generations after the inversion arises. This approach greatly simplifies the simulations because only the inversion frequency among Y chromosomes needs to be tracked. However, it is also a strong assumption where the time-dependent selection experienced by an inversion is modeled deterministically, while the inversion itself is subject to genetic drift due to random gamete sampling. Our approach qualitatively captures the effects of time-dependent selection on inversion fixation probability in large populations, where ephemeral indirect selection effects are most likely to be important, but ignores the potentially important effects of drift and linkage disequilibrium at selected loci in the chromosomal region spanned by inversions, which are best modeled by full Monte Carlo simulation (e.g., Jay et al., 2022; Lenormand & Roze, 2022, 2024).

We model the joint effects of time-dependent indirect selection and drift in a population with a total effective population size of N. Each generation, the inversion frequency is censused among the subpopulation of Y chromosomes prior to fertilization, following mutation and indirect selection. Following Connallon and Olito (2022), we use the deterministic change in fitness of males carrying either a non-inverted or inverted Y chromosome to predict the expected frequency of the inversion in the next generation. The realized frequency of the inversion in each generation is then simulated using pseudo-random binomial sampling during the gametic phase, with the number of Y chromosomes, N/2, representing the number of trials, and the expected frequencies of inverted Y chromosomes representing the binomial probability of sampling the inversion among pollen/sperm in the next generation. For each replicate simulation, the number of loci spanned by each new inversion was drawn from a Poisson distribution with mean and variance of $\bar{n}=n_{t\,o\,t}\,x$ and the number of deleterious alleles initially captured by a new inversion of length x was calculated as the sum of n binomial samples with mean probability of “success” equal to μ/(h⁢s). Hence, our simulations explicitly take into account the probability that an inversion of length x initially captures d deleterious mutations. Fixation probabilities were estimated from the outcomes of 200×N replicate simulations. We estimated the fixation probability of autosomal inversions using the same procedure but implementing multinomial pseudo-random sampling of adult genotypes (e.g., Charlesworth & Charlesworth, 2010, pp. 229–230). Additional simulation details are provided in Appendix D of the online supplementary material.

We focus our analysis on deleterious alleles with equal dominance coefficients of $h_{i}=h=0.25$, which corresponds roughly to the average dominance coefficient of deleterious mutations estimated from empirical studies (Manna et al. 2011; Agrawal & Whitlock 2012; reviewed by Billiard et al. 2021), and also corresponds to dominance values where the equilibrium assumptions underlying our model are well satisfied. We explore the effects of stronger recessivity in Appendix E of the online supplementary material (see Figure S17).

### Inversion fixation probabilities

**Figure 2:**
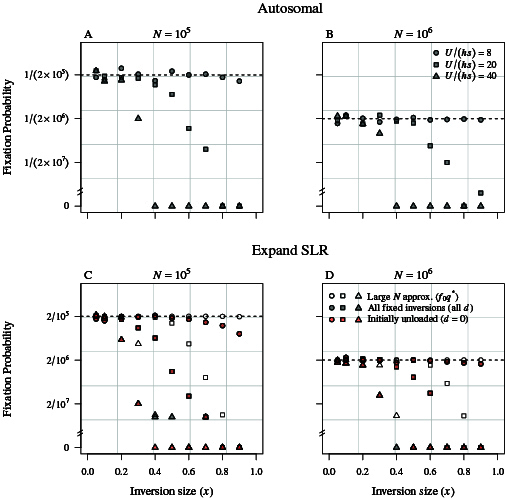
Fixation probabilities estimated from W–F simulations plotted as a function of inversion length for autosomal (A and B) and SLR-expanding inversions on Y chromosomes (C and D). Point shapes indicate different chromosome-arm-wide mutation rates relative to selection (i.e., different values of U), which influences the average deleterious mutation load carried by a standard-arrangement chromosome (U/(h⁢s)). Dashed horizontal lines indicate the corresponding expected fixation probability for a neutral variant for the same population size, and hence correspond to values of 1/⁢(𝟤𝑁) for autosomal inversions, and 𝟤/N for Y-linked inversions. In (C and D), we show fixation frequencies for all inversions (grey points), for only initially unloaded inversions (red points; i.e., the d=𝟢 inversion class), and the analytic approximation given by multiplying the probability that an inversion is initially mutation free by the effective initial frequency (unfilled points; i.e, $\text{𝖯𝗋}\,\left(\text{fix}|\text{𝑥}\right)\approx {\text{𝑓}}_{\text{𝟢}}\,{\text{𝑞}}^{*}$). *Note:* red points almost perfecty overlay grey points, but the few small discrepancies reflect rare additional fixations of initially (lightly) loaded inversions (where 𝑑>𝟢). Other parameter values were set to: h=0.25, s=0.01, and $n_{t\,o\,t}={\text{𝟣𝟢}}^{\text{𝟦}}$.

We examine the effect of inversion length on fixation probability because the fitness benefits and costs scale with inversion length for both autosomal and SLR-expanding inversions. The initial fitness benefit of capturing wild-type alleles at more loci increases with inversion length, but so does the probability of capturing more deleterious alleles and carrying a larger permanent deleterious load. For both autosomal and SLR-expanding inversions, these countervailing effects of inversion size cancel out when deleterious mutations are not strongly recessive and population sizes are sufficiently large, resulting in expected fixation probabilities that are independent of inversion length and approximately equal to the initial frequency of the inversion (Connallon & Olito, 2022). With partially recessive but still substantial fitness effects in heterozygotes and small population sizes, there is a fixation bias towards small inversions, which have a maximum fixation frequency among replicate simulations approximately equal to that of a typical neutral allele (1/(2⁢N) for autosomal inversions, 2/N for SLR-expanding ones; Figure 2A and C). In larger populations, the fixation frequency converges on the neutral expectation for all inversion sizes, particularly when there are fewer average deleterious mutations per standard chromosome arm (Figure 2B).

One key reason that inversions expanding the SLR on Y chromosomes behave so similarly to autosomal inversions is that, in both cases, inversions capturing even a single deleterious mutation are usually doomed to extinction (see Figure 1C and D). That is, despite the fact that SLR-expanding inversions have reduced homozygous expression of initially captured deleterious alleles relative to autosomal inversions (because males are never homozygous for a Y-linked inversion), the permanent load imparted to them by any captured deleterious alleles is still enough to effectively prevent them from going to fixation. Moreover, when deleterious allele frequencies on X and non-inverted Y chromosomes are assumed to remain at equilibrium, an initially unloaded SLR-expanding inversion experiences effectively the same time-dependent selection as an unloaded autosomal inversion (see Appendix B.4 of the online supplementary material). As a result, the same analytic approximations for the fixation probability hold as for autosomal inversions: the probability that an SLR-expanding inversion of length x is initially mutation-free is $f_{0}=e^{-U\,x/\left(h\,s\right)}$, while the fixation probability of an established mutation-free inversion is $q^{*}\approx q_{0}\,e^{U\,x/\left(h\,s\right)}+O\,\left(N^{-2}\right)$; the overall fixation probability is therefore approximately $\mathrm{Pr}\left(\text{fix}|x\right)\approx f_{0}\,q^{*}$, which converges to $q_{0}=2/N$ for a single-copy Y-linked inversion mutation when N is large and U is small (see Figure 2C and D; see also Appendix B in Connallon and Olito (2022) for derivation of the autosomal case).

## Discussion

The predictions of our models have several important implications. First, our deterministic models clarify that the notion proposed by several earlier studies—that linkage to the permanently heterozygous male-determining factor can shelter deleterious mutations from being expressed in homozygous form, thereby favoring recombination suppression—is an oversimplification. Deleterious alleles initially captured by an SLR-expanding inversion are not prevented from being expressed as homozygotes, as suggested by earlier verbal and mathematical arguments (Ironside, 2010; Jay et al., 2022). Rather, they are expressed as homozygotes at the frequency with which they occur on X chromosomes in ovules/eggs. This is the main difference between SLR-expanding and autosomal inversions, for which the homozygous expression of any captured deleterious alleles increases with the inversion frequency as more individuals become homozygous for the inverted haplotype, effectively preventing even lightly loaded inversions from ever fixing (Nei et al., 1967; Connallon & Olito, 2022). The deleterious load carried by lightly loaded SLR-expanding inversions is temporally dynamic but still permanent: initially beneficial inversions capturing even one mutation will eventually become deleterious (Figure 1). Moreover, in large populations, this deleterious load due to captured mutations effectively prevents all but initially mutation-free SLR-expanding inversions from ever fixing, resulting in similar evolutionary dynamics to autosomal inversions under many biologically plausible parameter conditions (Figure 2). Differences between the models only emerge when inversions rise to sufficiently high frequencies that selection on autosomal inversion homozygotes becomes important. This corresponds to conditions yielding high equilibrium frequencies of deleterious alleles: high mutation rates, weak selection, strongly recessive deleterious mutations, or some combination thereof (i.e., large U and/or small h⁢s; see Figures S4–15 of the online supplementary material).

Second, our W–F simulations confirm that similarly sized autosomal and SLR-expanding inversions subject to deleterious genetic variation should often have similar fixation probabilities (Figure 2). The reason for the similarity in behavior are as follows. The permanent deleterious load acquired by an inversion that captures even a single deleterious mutation prevents all but the initially mutation-free class of SLR-expanding inversions from ever fixing (at least when N is large), just as for autosomal inversions. Moreover, the time-dependent selection experienced by initially mutation-free SLR-expanding inversions is nearly identical to that experienced by a mutation-free autosomal inversion of comparable length, which explains the similarity in their dynamics. Whether our model predictions are consistent with empirical patterns of evolutionary strata size remains to be seen, but our results suggest that the length of SLR-expanding inversions may offer some insight into the selective processes driving their fixation. In particular, large evolutionary strata generally appear inconsistent with the inversion fixation probabilities expected for neutral inversions under partially recessive deleterious mutational variation, unless populations are large and the deleterious mutation rate is low.

It remains difficult to determine the relative importance of different hypotheses for suppressed recombination between sex chromosomes for several reasons. First, knowledge about the mutation rate and length distribution of new inversions in natural populations is limited, as is the number of well-described evolutionary strata, although genomic data are starting to shed light on these (Connallon & Olito, 2022; Charlesworth & Harkess, 2024). Second, our models make several simplifying assumptions. Generalizing to other cases is beyond the scope of the present paper, but important questions remain for future work. For example, allowing non-uniform distributions of dominance and selection coefficients will probably reduce the size of SLR-expanding inversions that ultimately become fixed, since this is the case for autosomal inversions (Connallon & Olito, 2022). Yet a few loci segregating for highly recessive deleterious alleles ($h_{i}\leq 0.05$) could have the opposite effect, though these would generally have strongly deleterious effects that would oppose this (Huber et al. 2018; Billiard et al. 2021; Olito & Charlesworth 2023). Lastly, our W–F simulations made several simplifying assumptions (discussed above in the Methods and Results section). Most notably, we combined deterministic changes in selection coefficients on inversions at mutation–selection balance with random sampling of haplotypes in each generation. Although the time-dependent indirect selection effects in our model will be most important in large populations, additional simulation studies are needed to determine at what population sizes (or population scaled selection parameter values) they become relevant (but see the discussion of Jay et al. 2022).

In addition to this study, two other recent theoretical studies have examined the fate of inversions evolving in the presence of deleterious mutational variation and suppressing recombination between sex chromosomes (Jay et al., 2022; Lenormand & Roze, 2022, 2024). Interestingly, these studies have come to very different conclusions regarding the fate of SLR-expanding inversions. Direct comparison between the different models’ predictions is difficult for a variety of reasons; including (i) the structure of the genetic systems modeled (inversions spanning relatively few (500 to $10^{4}$) to many ($2\times 10^{6}$) selected sites with constant selection parameters vs. fewer coding genes (500) with associated cis-/trans-regulators and an explicit underlying model of gene expression under stabilizing selection); (ii) the modeling approach (a combination of analysis, approximation, and W-F simulation vs. individual-based simulation); (iii) key model assumptions, including the presence/absence of genetic variation for reestablishing recombination; and (iv) the parameter values explored and emphasized. A detailed discussion of when these models are, or are not, comparable is beyond the scope of this study, but several key points deserve highlighting.

First, some explanation is needed for why our findings differ from those of Jay et al. (2022), who concluded from both deterministic modeling and individual-based simulations that Y-linked inversions expanding non-recombining regions are more likely to spread to high frequencies and go to fixation than autosomal inversions under broad parameter conditions. As we briefly explain below, our divergent conclusions are due to key differences in deterministic model assumptions, and in how inversion fixation probabilities were estimated from simulation data (see Olito & Charlesworth, 2023, for a detailed analysis and discussion). The deterministic models studied by Jay et al. (2022) assumed constant selection coefficients for inversions so that a Y-linked inversion initially capturing fewer than the population average number of deleterious mutations enjoyed a constant fitness advantage. As explained above, this assumption leads to overestimation of the selective advantage of new Y-linked inversions because it ignores the gradual loss of the initial fitness advantage due to the spread of new mutations at loci where a wild-type allele is initially captured.

The simulation results of Jay et al. (2022) focus on relatively small populations ($N=10^{3}$), and appear to show elevated fixation frequencies for SLR-expanding inversions relative to autosomal ones when either (i) selection is weak and mutations are partially recessive ($N_{e}\,s\leq 1$ and 0.1<h≤0.5) or (ii) selection is strong and deleterious mutations are completely recessive or nearly so ($N_{e}\,s>1$ and 0≤h≤0.1). Importantly, Jay et al. (2022) calculated inversion fixation frequencies conditioned on their survival beyond 20 generations. Conditional fixation frequencies do not provide an objective measure of the overall selective advantage accruing to new inversions evolving under the sheltering scenario, which can be quantified using the unconditional probability of fixation, averaging over all possible genetic backgrounds on which such an inversion can arise, and comparing with the neutral expectation (2/N for Y-linked inversions, 1/(2⁢N) for autosomal; Olito & Charlesworth 2023). Unconditional fixation frequencies calculated from Jay et al. (2022)’s simulation data show that when mutations were partially recessive (scenario i), Y-linked inversions generally fixed at frequencies that were neutral or less than neutral, which is consistent with our model predictions (Olito & Charlesworth, 2023). However, when selection was strong and deleterious mutations were completely recessive or nearly so (scenario ii), the simulations of Jay et al. (2022) show a robust pattern of elevated fixation frequencies for Y-linked inversions relative to neutrality, consistent with analytic approximations based on population genetics theory (Olito & Charlesworth, 2023). Importantly, Jay et al. (2022)’s results for highly recessive mutations correspond to and help illuminate the region of parameter space where our model assumptions break down.

Second, although the model(s) of Lenormand and Roze (2022; 2024) were originally designed to study Y recombination arrest under sex-specific regulatory evolution, and did not make comparisons with autosomal inversions, their results still shed some light on the robustness of our model predictions. In the absence of regulatory evolution (with only partially recessive deleterious mutations, similar to the senario we investigate here), Lenormand and Roze (2022; 2024) find higher fitness variance of large SLR-expanding inversions, but faster degradation of large inversions and a fixation bias toward small inversions when using parameter values corresponding to N⁢s=500 and a chromosome-wide deleterious mutation rate of U=0.1, results that are consistent with our model predictions (Figure 2; see Figures S4–S9 of the online supplementary material).

The studies of Lenormand and Roze (2022; 2024) also raise an interesting and relevant question: will SLR-expanding inversions contribute to long-term recombination suppression if there is genetic variation for reestablishing recombination (i.e., “reversions”)? Our results suggest that, even if they were lucky enough to fix, otherwise neutral SLR-expanding inversions initially capturing any deleterious alleles would be rapidly selected against and replaced by reversions due to the permanent deleterious load they carry. In fact, even initially mutation-free inversions are ultimately expected to be selected against due to selective interference and genetic degeneration (Lenormand & Roze, 2022, 2024; Bachtrog, 2008), but they should persist significantly longer than lightly loaded ones, and are therefore more likely to evolve dosage compensation or accumulate other structural rearrangements that might prevent reversion (see Lenormand & Roze, 2024, for a detailed discussion of these possibilities). Taking the possibility of reversions into account does not alter our familiar approximation for the combined probability that a new SLR-expanding inversion is initially mutation free, establishes, and goes to fixation ($\mathrm{Pr}\left(\text{fix}|x,r=0\right)\approx f_{0}\,q^{*}=2/N$). Our model predictions regarding inversion sizes should therefore remain valid in the presence of genetic variation for reestablishing recombination.

Notably, we have limited our attention to neutral inversions capturing deleterious mutations, without considering those with beneficial effects or capturing loci under sexually antagonistic selection that could cause linkage disequilibrium with the SLR. However, the presence of partially recessive deleterious genetic variation should influence the fixation probabilities of differently sized inversions, biasing the fixation probabilities toward smaller inversions in small populations or those with high mutation rates. We have also focused our analysis on single randomly mating populations at mutation selection balance and with selected loci initially at linkage equilibrium with the SLR, and evolving independently of one another. However, both inbreeding and prior linkage disequilibrium with the SLR feature prominently in previous sheltering hypotheses (Charlesworth & Wall 1999; Branco et al. 2017; see Appendix A of the online supplementary material for further details), and linkage disequilibrium between selected loci could also affect our model predictions. We briefly address the first two of these possibilities using mathematical models in Appendices F and G of the online supplementary material, where we show that they do not result in indirect positive selection for recombination-suppressing inversions. However, a more complete treatment of these questions is warranted.

Overall, our results indicate that when selection is strong relative to drift (N⁢s>1) and deleterious mutations are partially recessive (h⁢s≫μ), the sheltering hypothesis does not provide a plausible mechanism for elevated fixation probabilities of SLR-expanding inversions on proto-Y chromosomes compared to neutral expectations or autosomal inversions. The maximal fixation probability of such SLR-expanding inversions is roughly equal to that of a neutral gene, and drops below neutrality for some biologically plausible parameter conditions, especially for large inversions; a pattern that is mirrored by autosomal inversions. The exception is when most deleterious mutations are completely recessive or nearly so, in which case an otherwise neutral SLR-expanding inversion on a Y chromosome can enjoy significantly elevated fixation probabilities relative to neutrality (Jay et al., 2022; Olito & Charlesworth, 2023). Thus, under many biologically plausible conditions, the expansion of non-recombining regions on proto-Y chromosomes does not appear to be selectively favored due solely to the presence of partially recessive deleterious mutational variation.

## Supplementary material

Supplementary material is available online at *Evolution*.

## Supplementary Material

qpae060_suppl_Supplementary_Materials

## Data Availability

Computer code needed to reproduce the simulations and main figures is available on GitHub (https://github.com/colin-olito/shelteringOnSexChrom) and all versions of record are archived on Zenodo (doi:10.5281/zenodo.6361985).
