# Supplementary material for: Consequences of partially recessive deleterious genetic variation for the evolution of inversions suppressing recombination between sex chromosomes
Source: Evolution. 2024 Jun 10;78(8):1499–510. doi: 10.1093/evolut/qpae060 (PMC12372689; doi:10.1093/evolut/qpae060)
Supplement: qpae060_suppl_Supplementary_Materials [file qpae060_suppl_Supplementary_Materials.pdf]

Supporting Information (Appendices A–G) for: Consequences of partially recessive deleterious genetic variation for the evolution of inversions suppressing recombination between sex chromosomes. *Evolution*

Colin Olito*, Suvi Ponnikas[1], Bengt Hansson, Jessica K. Abbott

May 9, 2024

Department of Biology, Lund University, Lund 223 62, Sweden;
* Corresponding author; e-mail: colin.olito@gmail.com
[1] *Current address:* Ecology and Genetics Research Unit, 90014 University of Oulu, Finland;

Computer code needed to reproduce the simulations and main figures is available on GitHub (`https://github.com/colin-olito/shelteringOnSexChrom`) and all versions of record are archived on Zenodo (Olito et al. 2024)

Contents

Appendix A Previous heterozygote advantage and 'sheltering' hypotheses **3**
A.1 Ironside (2010) . . . 3
A.2 Ponnikas et al. (2018) . . . 3
A.3 Charlesworth & Wall (1999) . . . 4
A.4 Branco et al. (2017) . . . 5
A.5 Jay et al. (2022) . . . 6
A.6 Lenormand & Roze (2022, 2023) . . . 7

Appendix B Development of the deterministic model **8**
B.1 General 2-locus haplotype recursions . . . 8
B.2 Recursions when inversion captures either a wild-type or deleterious allele . . . 10
B.3 Multilocus recursion for inversion frequency . . . 11
B.4 Useful approximations . . . 11

Appendix C Autosomal Inversions **13**
C.1 Development of the exact recursions . . . 14

Appendix D Wright-Fisher simulations **16**

Appendix E Supplementary Figures **18**

Appendix F Revisiting the conjecture of Charlesworth & Wall (1999) **35**
F.1 Model . . . 35
F.2 Analysis . . . 35
F.3 Simulation results . . . 36

Appendix G Revisiting the verbal hypothesis of Branco et al. (2018): Inbreeding and Linkage **39**
G.1 Model . . . 39
G.2 Analysis . . . 40

Appendix A Previous heterozygote advantage and 'sheltering' hypotheses

The idea that linkage to the sex-determining gene(s) can cause a reduction in homozygous expression of partially recessive deleterious alleles at nearby loci has appeared several times in the literature. Unfortunately, the various verbal and mathematical models can be confusing because they have been formulated with different biological scenarios in mind (e.g., proto sex, neo-sex, and mating-type chromosomes), and have cited different theoretical studies as support. Below, we briefly summarize and quote relevant passages from key articles that have proposed or referenced this idea as either a verbal or mathematical model. We have added emphasis to particularly important passages in the quotations.

A.1 Ironside (2010)

To our knowledge, Ironside (2010) was the first to suggest that linkage to the dominant (*i.e.*, permanently heterozygous) sex-determining allele could cause a reduction in homozygous expression of deleterious mutations, and thereby indirect selection for suppressed recombination between sex chromosomes. Ironside's key contribution was to point out that the earlier models of Charlesworth and Wall (1999), which involved the formation of neo-sex chromosomes by nonreciprocal translocations in inbreeding populations, might be applicable to the evolution of suppressed recombination between sex chromosomes. In the subsection titled "Alternative hypotheses for the evolution of non-recombining regions", Ironside (2010) states:

> "Three alternative mechanisms are proposed to explain the spread of non-recombining regions on sex chromosomes ... The second is that suppression of recombination is selected because it prevents homozygosity of deleterious recessive genes, particularly in populations with inbreeding [33]."
> References: [33] Charlesworth and Wall (1999)

A second passage several paragraphs later elaborates on the hypothesis:

> "The hypothesis that chromosomal rearrangements spread through selection to prevent homozygosity of deleterious recessive genes at multiple loci was proposed by Charlesworth and Wall [33]. Their models demonstrate that, in populations with moderate levels of inbreeding, selection to maintain heterozygosity at two loci can favor the spread of neo-sex chromosomes generated by centric fusions or reciprocal translocations."

Overall, it is difficult to know exactly what Ironside (2010) was proposing. The relevant passages are not explicit about whether the hypothetical chromosomal rearrangement is selectively favored because it captures wild-type alleles, deleterious alleles, or a particular combination of both. Also, although the second passage clearly refers to neo-sex chromosome formation, the hypothesis was brought up in a discussion about the expansion of non-recombining regions by inversions surrounding the sex-determining loci on proto sex chromosomes. For example, Fig. 1 and Fig. 2 of Ironside (2010) very clearly depict inversions expanding the non-recombining region around a sex-determining locus, not neo-sex chromosome formation. It is not quite clear whether the mechanism is implied to work for the evolution of recombination suppression on proto sex chromosomes.

A.2 Ponnikas et al. (2018)

In their review of theories for the suppression of recombination between sex chromosomes (and empirical evidence supporting them), Ponnikas et al. (2018) refer to hypotheses involving "Heterozygote Advantage". The authors describe a very similar process to Ironside (2010) and cite Charlesworth and Wall (1999) and an older paper about translocations and mutational heterosis in completely selfing species (de Waal Malefijt and Charlesworth 1979):

Heterozygosity increases fitness by concealing recessive deleterious mutations and causing overdominance at functional loci [28]. Thus, it can be hypothesised that heterozygote advantage around a sex-determining gene in the heterogametic sex can favour recombination suppression (because less recombination means a larger heterozygote region). A challenge is however to explain how the sex-determining gene can be associated with a sufficient amount of inbreeding load (deleterious recessives) for the heterogametic sex to overcome the fitness costs of establishing genetic sex determination in the first place (induced by the skew in sex ratio, mentioned earlier; cf. [27]). A possible scenario is that the sex-determining gene is part of a larger rearrangement (reciprocal translocation or inversion) that captures a suite of loci carrying deleterious mutations, which then becomes fixed for heterozygosity in the heterogametic sex, which therefore experiences higher fitness due to heterozygote advantage. This implies that heterozygote advantage in principle is a possible agent of sex chromosome formation in evolutionary scenarios where inbreeding avoidance is favourable (Table 1).

References: [27] de Waal Malefijt and Charlesworth (1979), [28] Charlesworth and Wall (1999)

Ponnikas et al. (2018) are explicit in describing the linkage of loci with segregating recessive deleterious mutations to the sex-chromosomes but are ambiguous about which alleles ultimately become Y-linked. As in Ironside (2010), the authors clearly note that the models of Charlesworth and Wall (1999) refer to neo-sex chromosome evolution (and even provide a detailed schematic figure of neo-sex chromosome formation). However, it remains unclear whether they view this as a completely different process from the expansion of a non-recombining region around sex-determining loci by, for example, a chromosomal inversion. Their citation of de Waal Malefijt and Charlesworth (1979) in this context also introduces some confusion because this paper was motivated by the rather extreme example of heterozygosity of translocation complexes in the autogamous flowering plant genus *Oenothera*, and several key assumptions of the models they present are peculiar to obligately selfing hermaphrodites.

A.3 Charlesworth & Wall (1999)

This important paper described the invasion of centric fusions or translocations causing a single autosomal locus with heterozygote advantage to become completely linked to either the Y or X chromosome in a partially inbreeding population (they modeled partial full-sib mating). Here, we give a brief overview of their hypotheses and models, but refer readers to the original article for further details. The authors made two statements suggesting that their results might be relevant to scenarios involving selected loci under deleterious mutation pressure. In the last paragraph of the introduction, the authors explain that their choice of model was a practical one:

> "There is therefore a need to investigate quantitatively the question of the nature of selection for neo-X or neo-Y chromosomes in inbreeding populations. The causes of inbreeding depression and heterosis are still a matter for debate, but it seems likely that both deleterious mutations and alleles maintained by heterozygote advantage play a role, with the former probably being more important (Crow 1993; Charlesworth 1998). It is, however, much simpler to model the case of a single locus with heterozygote advantage rather than a multi-locus model of mutation and selection, and this is the subject of the present paper. The results confirm that there is, indeed, often selection for a fusion or translocation between a sex chromosome and an autosome in a partially inbreeding species when there is heterozygote advantage."

In the final sentences of the Discussion, the authors make the following extremely cautious conjecture regarding the relevance of their findings to the case of deleterious mutations:

> "Second, we have assumed that heterosis is caused by a single locus with heterozygote advantage. Since homozygosity for a chromosome segment in an inbreeding population is likely to be associated with reduced fitness in much the same way as homozygosity for an allele at a locus with heterozygote advantage,

the present study probably provides a rough guide to what is likely to be true in the case of mutational heterosis (Charlesworth 1991). A detailed investigation of this case by computer simulations is needed to verify this conjecture."

Subsequent references to the models of Charlesworth and Wall (1999) by other authors to explain either neo-sex chromosome formation *or* recombination suppression between proto sex chromosomes have introduced a couple points of confusion. First, and most importantly, because the original models used a single locus under overdominant selection, all subsequent references to them as support for the idea that linkage to the sex-determining locus can reduce homozygote expression of deleterious alleles is based on the conjecture rather than the model predictions. Second, despite this issue of referencing, the models of Charlesworth and Wall (1999) are still relevant to the process of recombination suppression between proto sex chromosomes. Specifically, their model is mathematically equivalent to a model of the expansion of non-recombining region surrounding the sex-determining loci to capture a selected locus under heterozygote advantage, provided there is free recombination between the sex-determining and selected loci on the standard arrangement chromosomes. Despite the confusion surrounding Charlesworth & Wall's conjecture, the scenarios of neo-sex chromosome formation and expansion of the non-recombining region around the sex-determining loci by an inversion (or other recombination modifier) are conceptually and mathematically linked. Unfortunately, neither of the above review articles made this connection, or the distinction between the scenarios, explicit.

Overall, it seems clear that Charlesworth & Wall's conjecture regarding the applicability of their model results to the case of mutational heterosis deserves closer scrutiny. In Appendix F, we revisit the models of Charlesworth and Wall (1999) and expand them to address the case of an inversion linking multiple selected loci under deleterious mutation pressure to the dominant male-determining allele on a proto sex chromosome. Our results indicate that the conjecture, upon which the later reviews and citing articles have based their argumentation, does not appear to work.

A.4 Branco et al. (2017)

Branco et al. (2017) proposed a very similar hypothesis to those of Ironside (2010) and Ponnikas et al. (2018), but approached the idea from a different perspective: their study focused on the evolution of recombination suppression between haploid mating-type chromosomes in some fungi. However, Branco et al. (2017) are explicit in stating that deleterious alleles must become linked to mating-type or sex-determining loci. They propose the following hypothesis in their Supplementary Material:

> "Another evolutionary explanation for suppressed recombination on fungal mating-type chromosomes, that could also apply to sex chromosomes, involves linkage of deleterious alleles to the mating-type loci, favoring permanent sheltering in an heterozygous state, as has been theoretically modeled [20, 21]. This may arise specifically in sex and mating-type chromosomes if recombination frequency gradually decreases from the non-recombining region into the PAR, so that partial linkage (linkage disequilibrium) to mating-type or sex-determining genes makes selection against recessive deleterious alleles less efficient. This would allow deleterious alleles to increase in frequency in these PAR edges at the margin of the non-recombining region. Rare recombination events would then generate individuals homozygous for deleterious alleles and could therefore be selected against. Complete linkage of these PAR margins in disequilibrium with mating type may thus be favorable and selected for (Fig. S1A). Permanent sheltering would thus be more easily achieved than purging if recombination is rare at the PAR margins. Further theoretical models are needed to explore the general conditions under which such a mechanism can generate evolutionary strata."
>
> References: [20] Antonovics & Abrams (2004); [21] Johnson et al. (2001).

Branco et al. (2017)'s verbal model also introduces confusion because the models of mating-type chromosomes they cite involve a variety of different assumptions that, while reasonable for some fungi species with haploid sex-

determination, do not hold for species with diploid genetic sex-determination (e.g., intra-tetrad mating). Moreover, this verbal model introduces a new complicating factor that was not present in the models of Charlesworth and Wall (1999): genetic linkage between the mating-/sex-determining loci and load loci prior to the evolution of arrested recombination between them. In their Fig. S1A, they explicitly depict deleterious mutations becoming linked to mating-type alleles.

In Appendix G, we address the potential for linkage and identity disequilibrium between the sex-determining and selected loci to influence the invasion of a recombination modifier suppressing recombination between them.

A.5 Jay et al. (2022)

In a theoretical study using both deterministic models and individual-based simulations, Jay et al. (2022) presented the most explicit hypothesis to date involving the evolution of recombination suppression beteen sex chromosomes due to 'sheltering' of partially recessive deleterious alleles on the Y chromosome. In this article, the authors explicitly proposed that inversions expanding the sex-linked region on a Y chromosome that capture partially recessive deleterious alleles will enjoy a selective advantage because those deleterious alleles will be permanently sheltered in heterozygous form on the Y. They state the hypothesis verbally in the paper and attempt to formalize it as a mathematical and simulation model. The verbal argument is articulated in the introduction as follows:

> Now, consider an inversion that, by chance, captures a permanently heterozygous allele, such as the male-determining allele in an XY system. If this Y-linked inversion captures fewer deleterious variants than the population average, it should increase in frequency without ever suffering the deleterious consequences of having its load expressed. The recessive deleterious mutations captured by the sex-linked inversion are, indeed, fully associated with the permanently heterozygous, male-determining allele, and will, therefore, never occur as homozygotes. Unlike autosomal inversions, Y-linked inversions retain their fitness advantage with increasing frequency (Figure 1C). Hence, less heavily loaded Y-linked inversions would be expected to spread, becoming fixed in the population of Y chromosomes, resulting in a suppression of recombination between the X and Y chromosomes in the region covered by the inversion.

The deterministic model presented in the paper formalizes this verbal model by ignoring recurrent mutations on inversions once they arise. That is, they assumed that inversions experience constant fitness effects that are completely determined by the number of deleterious mutations they initially capture. The key assumption is stated in the main text as follows:

> The deterministic increase in frequency of an inversion (*I*) on an autosome or capturing an XY-like sex-determining locus can easily be determined with a two-locus two-allele model ... Based on this model, and initially assuming that inverted and non-inverted segments no longer accumulate deleterious mutations after their formation (i.e. W_{II}, W_{NI} and W_{NN} are fixed parameters), we simulated the evolutionary trajectory of inversions on autosomes and of inversions capturing the male-determining allele on the Y chromosome under a wide range of parameter values.

Hence, the fitness effects of inversions in their deterministic model do not take into account either (*i*) the accumulation of new mutations on descendent copies of the inversion at sites where they initially captured a wild-type allele, or (*ii*) changes in deleterious allele frequencies on X chromosomes at sites where the inversion initially captures a deleterious mutation. Under these assumptions, a new SLR-expanding inversion will indeed be permanently beneficial if it captures fewer than the average number of deleterious alleles in the chromosomal segment it spans.

However, in their simulations Jay et al. (2022) did explicitly model recurrent mutations on inversions. The simulations focused on finite populations of $N = 10^3$ individuals in which drift, indirect selection, and linkage disequilibrium could jointly influence inversion frequency dynamics. However, the analysis and interpretation of the simulation results introduced significant confusion. Olito and Charlesworth (2023) present a detailed analysis and discussion of Jay et al. (2022)'s simulation data, and how it was presented and misinterpreted. In short, Jay et al. (2022)'s published estimates of fixation probabilities for Y-linked inversions were in fact conditional fixation frequencies calculated after excluding inversions that were lost in the first 20 generations. This generates a substantial bias towards high fixation probabilities and obscures comparisons with neutrality. If the unconditional fixation frequencies are calculated using all of Jay et al. (2022)'s simulation runs, the estimates of fixation probabilities are close to neutral expectation for most parameter sets explored for both autosomal and Y-linked inversions, unless deleterious mutations are close to being completely recessive, in which case Y-linked inversions did have elevated fixation probabilities.

Jay et al. (2022) also provided supplementary simulation results for larger populations ($N = 10^4$), which gave mixed results. For larger population sizes, fixation of autosomal inversions becomes exceedingly rare. This appears to be due to inadequate replication of the simulations. Prior theory focusing on large populations (Connallon and Olito 2020) predicts a fixation rate of $1/(2N)$ for autosomal inversions when taking into account time-dependent selection due to accumulation of deleterious mutations. For $N = 10^4$, this corresponds to a fixation probability of $1/(2 \times 10^4)$. However, because only 10^4 replicate simulations were performed, only $10^4/(2 \times 10^4) = 0.5$ fixation events are expected on average for this level of replication, consistent with the supplementary figures. For Y-linked inversions, the elevated fixation frequencies when mutations were highly recessive ($h \leq 0.1$) were robust, but for less recessive mutations ($h > 0.1$) the results are still mostly indistinguishable from neutrality and the level of replication is still too low to precisely estimate such small fixation probabilities.

As explained in Olito and Charlesworth (2023), the findings of Jay et al. (2022)'s simulation study must be carefully reexamined after calculating the unconditional fixation frequencies, and interpreted with caution because of low replication (and therefore low precision of the estimated unconditional fixation frequencies). Nevertheless, their finding that SLR-expanding Y-linked inversions have elevated fixation probabilities relative to neutrality when deleterious mutations are completely recessive or very close to it is robust, and consistent with analytic approximations based on population genetics theory.

A.6 Lenormand & Roze (2022, 2023)

A recent simulation study of Y recombination arrest and sex-specific regulatory evolution inadvertently shed light on "sheltering hypotheses", and provides some indication of the robustness of the major assumptions in our Wright-Fisher simulations. Lenormand and Roze (2022) used individual based simulations of SLR-expanding inversions on still-recombining sex chromosomes. Their focus was to study the consequences of sex-specific regulatory evolution subsequent to the fixation of SLR-expanding inversions, and they included additional assumptions (such as the possibility that fixed inversions were replaced by "reversions" to the original chromosomal arrangement). However, they assumed that these "lucky inversions" initially fixed while infuenced by indirect selection due solely to segregating partially recessive deleterious mutations (see Lenormand and Roze 2022 Supplementary Material). They explain in the main text of their article:

> To understand the dynamics of sex chromosome evolution in our model, first consider the case where the cis and trans regulators do not mutate. In this case, all inversions on the Y are eventually reversed and lost. This occurs in two steps: First, an inversion appears on a given Y and "freezes" a segment of the chromosome. If by chance this Y carries relatively few or milder deleterious mutations, this "lucky" inversion

has a selective advantage. Consequently, it tends to fix among Y chromosomes, causing recombination suppression in this portion of the sex chromosomes. Larger inversions are overrepresented among these lucky inversions, as they contain more genes and exhibit a larger fitness variance (25) (fig. S1A). Once fixed, these Y chromosomes start accumulating deleterious mutations as a result of selective interference. Fitness declines faster for larger inversions because of stronger selective interference (fig. S1B).

In their supplement (fig. S1A,), they show both that larger inversions do indeed have larger fitness variance, but also degrade faster. However, the degredation is due to a combination of the accumulation of deleterious mutations as deleterious allele frequencies return to equilibrium, and selective interverence. Yet, they also found that smaller inversions had a higher probability of being fixed and subsequently stabilized (their fig. S2C).

In an even more recent article, which originally appeared as a preprint Lenormand and Roze (2024) used the same modeling framework to explicitly simulation the 'sheltering' scenario by omitting sex-specific trans-regulators. They showed that SLR-expanding inversions can indeed go to fixation, but made no comparisons with the fixation frequency expected under neutrality or with comparable autosomal inversions. Rather, they emphasized the importance of genetic variation for reestablishing recombination after inversions had fixed (so called 'reversions'), which we discuss briefly in the main text.

Appendix B Development of the deterministic model

To model the evolution of new SLR-expanding inversions, we need to derive recursions describing per-generation gene frequency changes for two categories of loci: (1) loci where the inversion intially captures a wild-type allele and (2) those where it captures a deleterious allele, within four different chromosome classes, (1) X's in ovules/eggs, (2) X's in pollen/sperm, (3) non-inverted Y's, and (4) inverted Y's. Under our assumption that the selected loci are unlinked in all but the inverted Y chromosome class, we can derive these recursions from a simple 2-locus PAR model involving a sex-determining locus and a second selected locus under deleterious mutation pressure. We then use the resulting recursions to formulate a model with multiple selected loci at linkage equilibrium. All of the same model assumptions noted in the main text still apply (*i.e.*, large population size, discrete generations, etc.)

B.1 General 2-locus haplotype recursions

Consider the following two-locus genetic system: one locus determines whether a chromosome is considered X or Y, with XX individuals being female, and XY individuals being male; and a second locus that may be linked to the sex-determining region and is subject to natural selection. At the selected locus, the wild-type allele, A, mutates to a deleterious variant, a, at a rate μ per chromosome per generation (we ignore backmutation from $a \to A$). Recombination between the two loci occurs at a rate r per meiosis. Generations are discrete, and the population size is assumed to be large enough that drift is negligible. The life cycle proceeds: mutation → selection → random mating.

There are three relevant female genotypes at the selected locus: AA, Aa, and aa, with frequencies denoted x_1, x_2, and x_3, and general fitness expressions at selection denoted $w_{f,1}$, $w_{f,2}$, $w_{f,3}$. However, there are eight relevant genotypes for males: AA, AA^I, Aa (cis-), Aa^I (cis-), aA (trans-), aA^I (trans-), aa, and aa^I, with frequencies y_1, y_1^I, y_{2c}, y_{2c}^I, y_{2t}, y_{2t}^I, y_3, and y_3^I, and relative fitness terms $w_{m,1}$, $w_{m,2}$, $w_{m,3}$. Note that male heterozygote genotype labels indicate whether the A allele is located on the X chromosome (y_{2c}), or on the Y chromosome (y_{2t}), and "I" superscripts denote inverted haplotypes. It is assumed that recombination is completely suppressed between inverted and non-inverted chromosomes.

We denote the frequency of each of the relevant haplotypes as follows:

$X_{A,Ov}$: the frequency of proto-X chromosomes carrying the A allele in ovules/eggs,

$X_{a,Ov}$: the frequency of proto-X chromosomes carrying the a allele in ovules/eggs,

$X_{A,Sp}$: the frequency of proto-X chromosomes carrying the A allele in pollen/sperm,

$X_{a,Sp}$: the frequency of proto-X chromosomes carrying the a allele in pollen/sperm, and

Y_A, Y_A^I, Y_a, Y_a^I : the frequency of inverted & non-inverted proto-Y chromosomes carrying each allele in pollen/sperm.

The frequency of the three female genotypes after random mating are equal to:

$$
\begin{aligned}
x_1 &= X_{A,Ov}X_{A,Sp} \\
x_2 &= X_{A,Ov}X_{a,Sp} + X_{a,Ov}X_{A,Sp} \\
x_3 &= X_{a,Ov}X_{a,Sp}
\end{aligned}
$$

and the frequency of the eight relevant male genotypes after random mating are:

$$
\begin{aligned}
y_1 &= X_{A,Ov}Y_A \\
y_1^I &= X_{A,Ov}Y_A^I \\
y_{2c} &= X_{A,Ov}Y_a \\
y_{2c}^I &= X_{A,Ov}Y_a^I \\
y_{2t} &= X_{a,Ov}Y_A \\
y_{2t}^I &= X_{a,Ov}Y_A^I \\
y_3 &= X_{a,Ov}Y_a \\
y_3^I &= X_{a,Ov}Y_a^I
\end{aligned}
$$

The genotypic frequencies among females after mutation are:

$$
\begin{aligned}
x_1^u &= x_1(1-2\mu) \\
x_2^u &= x_2(1-\mu) + 2x_1\mu \\
x_3^u &= x_3 + x_2\mu
\end{aligned}
\tag{B1}
$$

and among males:

$$
\begin{aligned}
y_1^u &= y1(1-2\mu) \\
y_1^{I,u} &= y_1^I(1-2\mu) \\
y_{2c}^u &= y_{2c}(1-\mu) + y_1\mu \\
y_{2c}^{I,u} &= y_{2c}^I(1-\mu) + y_1^I\mu \\
y_{2t}^u &= y_{2t}(1-\mu) + y_1\mu \\
y_{2t}^{I,u} &= y_{2t}^I(1-\mu) + y_1^I\mu \\
y_3^u &= y_3 + (y_{2c} + y_{2t})\mu; \\
y_3^{I,u} &= y_3^I + (y_{2c}^I + y_{2t}^I)\mu
\end{aligned}
\tag{B2}
$$

The haplotype frequencies among female gametes (ovules/eggs) after selection and meiosis are:

$$X'_{A,Ov} = \left(x^u_1 w_{f,1} + \frac{x^u_2 w_{f,2}}{2}\right)\Big/\overline{w}_f$$
$$X'_{a,Ov} = \left(x^u_3 w_{f,3} + \frac{x^u_2 w_{f,2}}{2}\right)\Big/\overline{w}_f \tag{B3}$$

where $\overline{w}_f = x^u_1 w_{f,1} + x^u_2 w_{f,2} + x^u_3 w_{f,3}$. The corresponding haplotype frequencies among pollen/sperm are:

$$X'_{A,Sp} = \left(y^u_1 w_{m,1} + y^{I,u}_1 w_{m,1} + y^u_{2c} w_{m,2}(1-r) + y^{I,u}_{2c} w_{m,2} + y^u_{2t} w_{m,2} r\right)\big/\overline{w}_m$$
$$X'_{a,Sp} = \left(y^u 2c w_{m,2} r + y^u_{2t} w_{m,2}(1-r) + y^{I,u}_{2t} w_{m,2} + y^u_3 w_{m,3} + y^{I,u}_3 w_{m,3}\right)\big/\overline{w}_m$$
$$Y'_A = \left(y^u_1 w_{m,1} + y^u_{2c} * w_{m,2} r + y^u_{2t} w_{m,2}(1-r)\right)\big/\overline{w}_m$$
$$Y^{I\prime}_A = \left(y^{I,u}_1 w_{m,1} + (y^{I,u}_{2t} w_{m,2})\right)\big/\overline{w}_m$$
$$Y'_a = \left(y^u_{2c} w_{m,2}(1-r) + y^u_{2t} w_{m,2} r + y^u_3 w_{m,3}\right)\big/\overline{w}_m$$
$$Y^{I\prime}_a = \left(y^{I,u}_{2c} w_{m,2} + y^{I,u}_3 w_{m,3}\right)\big/\overline{w}_m \tag{B4}$$

where $\overline{w}_m = y^u_1 w_{m,1} + y^{I,u}_1 w_{m,1} + y^u_{2c} w_{m,2} + y^{I,u}_{2c} w_{m,2} + y^u_{2t} w_{m,2} + y^{I,u}_{2t} w_{m,2} + y^u_3 w_{m,3} + y^{I,u}_3 w_{m,3}$.

Note that the recombination rate, r, does not enter into the recursions for X chromosomes in females. This is because recombination events in females do not change the frequency of X_A or X_a haplotypes among ovules. The following substitutions allow us to simplify things: $X_{A,Ov} = 1 - X_{a,Ov}$, $X_{A,Sp} = 1 - X_{a,Sp}$, $Y_A = 1 - Y_a - Y^I_A - Y^I_a$. Also, because we assume the selected locus is initially in linkage equilibrium with the sex-linked region, the rate of recombination per meiosis between the two loci will be $r = 1/2$. We can now describe the frequency dynamics with a system of 5 simplified haplotype recursions: $X'_{a,Ov}$, $X'_{a,Sp}$, Y'_a, Y'^I_A, and Y'^I_a.

Ultimately, we want to be able to express our recursions in terms of the frequency of deleterious alleles in each of the different chromosome classes: X chromosomes in ovules/eggs, X chromosomes in pollen/sperm, non-inverted Y chromosomes, and inverted Y chromosomes. To do this, we must transform our haplotype recursions onto a new coordinate system by introducing 3 new variables: Y_I, the frequency of the inversion among Y chromosomes; q_Y, the relative frequency of the deleterious a allele at the selected locus among non-inverted Y chromosomes; and q_{Y^I}, the relative frequency of the deleterious a allele at the selected locus among inverted Y chromosomes. Noting that $Y_I = Y^I_A + Y^I_a$ and the four frequencies Y_A, Y_a, Y^I_A, Y^I_a sum to one, we can make the following substitutions: $q_Y = Y_a/(1-Y_I)$, $p_Y = Y_A/(1-Y_I)$, $q_{Y^I} = Y^I_a/Y_I$, and $p_{Y^I} = Y^I_A/Y_I$, and define the new recursions:

$$q'_Y = Y'_a/(1-Y'_I)$$
$$p'_Y = 1 - Y'_I - Y'_a$$
$$q'_{Y^I} = Y'^I_a/Y'_I$$
$$p'_{Y^I} = Y'_I - Y'^I_a$$
$$Y'_I = (Y'^I_A + Y'^I_a)/(Y'_A + Y'_a + Y'^I_A + Y'^I_a) \tag{B5}$$

For consistency of notation, we relabel $X_{a,Ov} = q_{X_f}$ and $X_{a,Sp} = q_{X_m}$. We can now describe the frequency dynamics with the following recursions: q'_{X_f}, q'_{X_m}, q'_Y, q'_{Y^I}, and Y'_I. We develop the multilocus recursion for Y'_I below in B.3.

B.2 Recursions when inversion captures either a wild-type or deleterious allele

A new single-copy inversion mutation spanning both the sex-determining and selected loci will capture either a wild-type (A) or deleterious (a) allele at the selected locus. As explained in the main text, the allele frequency dynamics

among each of the chromosome classes will differ depending on which allele the inversion captures. In the case where a new (rare) inversion captures a wild-type allele, the above recursions can be used to describe the resulting per-generation allele frequency changes in each chromosome class. We can model the case where a new inversion captures the deleterious a allele by substituting $Y^I_A = 0$ into the above recursion system. In this case all descendent copies of the inversion will carry that allele (*i.e.*, $q_{Y^I} = 1$ and $p_{Y^I} = 0$ for all t future generations).

The full two-locus recursions for q'_{X_f}, q'_{X_m}, q'_Y, and q'_{Y^I} when the inversion captures either a wild-type or deleterious allele are unwieldy, and are presented in the accompanying Mathematica notebook file in the Online Supplementary Material.

B.3 Multilocus recursion for inversion frequency

To describe the frequency dynamics of an inversion that captures any number of unlinked selected loci, we have to make several simplifying assumptions (outlined in the main text). In brief, we assume the inversion captures n loci, and that the mutation selection parameters are constant across all captured loci (*i.e.,* $\mu_i = \mu$, $s_i = s$, and $h_i = h$). We can then categorize the PAR loci spanned by the inveresion by which allele is captured. The gene frequencies at each of the n loci at time t can be described using the following notation: $q^D_{X_f,t}$, $q^D_{X_m,t}$, $q^D_{Y,t}$, $q^D_{Y^I,t}$, and $q^W_{X_f,t}$, $q^W_{X_m,t}$, $q^W_{Y,t}$, $q^W_{Y^I,t}$, where q refers to the deleterious allele frequency, and D and W superscripts denote which allele was initially captured by the inversion at the ith locus (D denotes loci where a deleterious allele was initially captured, W denotes loci where a wild-type allele was initially captured). To simplify notation, we use the convention $p^{\cdot}_{\cdot,t} = 1 - q^{\cdot}_{\cdot,t}$.

We can now define the following recursion for Y'_I that takes into account the fitness effects of the alleles it captures at all n loci:

$$Y^I_{(t+1)} = Y^I_t \left[\Big(1 - s\big(hp^D_{X_f,t} + q^D_{X_f,t}\big)\Big)^d \Big(1 - s\big(h(p^W_{Y^I,t}q^W_{X_f,t} + q^W_{Y^I,t}p^W_{X_f,t}) + q^W_{Y^I,t}q^W_{X_f,t}\big)\Big)^{n-d} \right] \Big/ \overline{w}^Y \tag{B6}$$

where

$$\begin{aligned}\overline{w}^Y = Y^I_t &\left[\Big(1 - s\big(hp^D_{X_f,t} + q^D_{X_f,t}\big)\Big)^d \Big(1 - s\big(h(p^W_{Y^I,t}q^W_{X_f,t} + q^W_{Y^I,t}p^W_{X_f,t}) + q^W_{Y^I,t}q^W_{X_f,t}\big)\Big)^{n-d} \right] \times \\ (1 - Y^I_t) &\left[\begin{array}{c} \Big(1 - s\Big(h\big(p^D_{X_f,t}q^D_{Y,t} + q^D_{X_f,t}p^D_{Y,t}\big) + q^D_{X_f,t}q^D_{Y,t}\Big)\Big)^d \\ \Big(1 - s\Big(h\big(p^W_{X_f,t}q^W_{Y,t} + q^W_{X_f,t}p^W_{Y,t}\big) + q^W_{X_f,t}q^W_{Y,t}\Big)\Big)^{n-d} \end{array} \right]\end{aligned} \tag{B7}$$

Noting that $q^D_{Y^I,t+1} = 1$. Equations (B6) and (B7) are presented as equations (1) and (2) in the main text.

B.4 Useful approximations

Prior to any recombination suppression between the sex chromosomes, the dynamics of deleterious mutations are effectively identical to those on the autosomes (provided that selected loci are segregate independently from the SLR). Assuming weak selection and mutation, the approximate change in frequency of a deleterious mutation in continuous time is (after Nei et al. 1967):

$$\frac{\partial q}{\partial t} = Ux - hsq_t. \tag{B8}$$

The general solution to this differential equation is

$$q_t = \frac{Ux}{hs}(1 - e^{-hst}). \tag{B9}$$

From this result, we can derive the fitness expressions for an initially mutation-free SLR-expanding inversion on the Y inversion due to accumulation of deleterious mutations. Under our stated assumptions, the inversion does not disrupt the function of any genes that it spans, and only serves to suppress recombination between the X and Y chromosomes. In this case, inversion-bearing males will have fitness equivalent to a heterozygote in the Autosomal model. It is important to note that, despite the fact that the inverted chromosomal segment is effectively haploid (exists only as a single-copy in males), the relative fitness of non-inversion bearing males still depends on the dominance coefficient for two reasons: (1) because we assume the genes in the chromosomal region spanned by the inversion are still functional; and (2) because the X and Y chromosomes still recombine in the region spanned by the inversion in N_Y/N_X males. Standardizing relative to the I_Y/N_X genotype, we have:

Generation	Fitness of I_Y/N_X	Fitness of N_Y/N_X
0	e^{-Ux}	e^{-2Ux}
1	$e^{-Ux(2-e^{-hst})}$	e^{-2Ux}
t (relative to N_Y/N_X)	$e^{Uxe^{-hst}}$	1
t (approx.)	$1+Uxe^{-hst}$	1

Note: Our approximation for the initial relative fitness of an unloaded inversion appears here, in the above table. With these time-dependent fitness expressions in hand, we can solve for the general solution for the frequency of the inversion in discrete time, which we denote q_t here (and only here) for consistency with Connallon and Olito (2020). Beginning with the recursion

$$q' = \frac{qe^{-Ux(2-e^{-hst})}}{(1-q)e^{-2Udx}+qe^{-Ux(2-e^{-hst})}}, \tag{B10}$$

We can rearrange to track the ratio of the expected frequencies of deleterious and wild-type alleles:

$$\frac{q'}{1-q'} = \frac{q}{(1-q)}e^{e^{-hst}Ux} \tag{B11}$$

Because the change in the ratio of inversion to standard arrangement chromosome frequencies will be multiplicative over time, we can simplify:

$$\begin{aligned}
\frac{q'}{1-q'} &= \frac{q_0}{(1-q_0)}\prod_{i=0}^{t-1} e^{e^{-hsi}Ux} \\
&= \frac{q_0}{(1-q_0)}e^{\sum_{i=0}^{t-1} e^{-hsi}Ux} \\
&= \frac{q_0}{(1-q_0)}e^{Ux\sum_{i=0}^{t-1} e^{-hsi}} \\
&= \frac{q_0}{(1-q_0)}e^{\frac{Ux(1-e^{-hst})}{1-e^{1-hs}}}.
\end{aligned} \tag{B12}$$

Solving for q_t gives the general discrete time solution for the change in inversion frequency:

$$q_t = \frac{e^{\frac{Ux(1-e^{-hst})}{1-e^{-hs}}}q_0}{1-q_0+e^{\frac{Ux(1-e^{-hst})}{1-e^{-hs}}}q_0} \tag{B13}$$

This is the discrete time general solution for the change in frequency of an SLR-expanding inversion used in Fig. 1 of the main text, and in Supplementary figures.

From Eq(B13), we can find the long-term deterministic frequency of the inversion, denoted q^*, by taking the limit:

$$q^* = \lim_{t\to\infty} \frac{e^{\frac{Ux(1-e^{-hst})}{1-e^{-hs}}} q_0}{1 - q_0 + e^{\frac{Ux(1-e^{-hst})}{1-e^{-hs}}} q_0} = \frac{e^{\frac{Ux}{1-e^{-hs}}} q_0}{1 - \left(1 - e^{\frac{Ux}{1-e^{-hs}}}\right) q_0},$$

which, to first order in N, is

$$q^* \approx q_0 e^{\frac{Ux}{hs}} + O(N^{-2}) \tag{B14}$$

where $q_0 = 2/N$ for a single-copy inversion mutation. This is the approximation presented in the subsection *Deterministic frequency dynamics*, and which we use as a benchmark in Fig. 1 of the main text, and in the Supplementary Figures. Corresponding approximations for Autosomal inversions have been derived previously by Connallon and Olito (2020) (see their Appendix B), and follow very closely the results obtained here, but using the appropriate initial frequency for a single-copy mutation for an autosomal gene, $q_0 = 1/2N$.

Finally, the autosomal model of Connallon and Olito (2020) made the simplifying assumption that deleterious alleles on non-inverted chromosomes remain at equilbrium frequencies during the process of inversion establishment. Making the same equilibrium approximation for SLR-expanding inversions on the Y chromosome (i.e., that deleterious mutations on X's and non-inverted Y's remain at equilibrium) enables us to simplify the full model down to a single 'haploid' model of inversion frequency dynamics among Y chromosomes. Specifically, we can express the per-generation change in inversion frequency as

$$Y_I' = Y_I w_{Y_I} / \overline{w}, \tag{B15}$$

where the fitness of inverted and non-inverted Y chromosomes are

$$w_{Y_I} = \left(\hat{q}(1-s) + (1-\hat{q})(1-sh)\right)^d \left(1 - \mu(2 - e^{-sht})\right)^{n-d} \tag{B16a}$$

$$w_Y = (1-2\mu)^n, \tag{B16b}$$

respectively, and $\overline{w}$ is the sum of Eqs(B16). We compare the predictions of this haploid equilibrium approximation with the full set of exact recursions in Supplementary figures S4–S9, where it can be seen that the haploid model performs very well provided that deleterious mutations are not strongly recessive, and mutation rates are not large.

Appendix C Autosomal Inversions

The full set of deterministic recursions for SLR-expanding inversions on Y chromosomes (see Appendix B) allows for feedback between the inversion frequency and the frequency of deleterious alleles on X chromosomes. This feedback was not possible in the model of Autosomal inversions described by Connallon and Olito (2020). Specifically, Connallon and Olito (2020) made the simplifying assumption that deleterious allele frequencies on non-inverted chromosomes remained at the equilibrium frequency of $\hat{q} = u/(hs)$. Here, we develop a corresponding full set of exact recursions for Autosomal inversions which allows for feedback between the inversion frequency and the frequency of deleterious alleles on non-inverted chromosomes. In fact, Nei et al. (1967) developed exact recursions for the special case where all deleterious mutations are fully recessive (i.e., where $h = 0$). Below, we extend their model to accommodate partially recessive deleterious mutations ($0 < h < 0.5$).

C.1 Development of the exact recursions

Following the derivation of Nei et al. (1967), the deterministic changes in the genetic composition of the population can be described in terms of the gene frequencies at each of the loci spanned by the inversion, and the inversion frequency. As we did for SLR-expanding inversions, we also denote loci at which the inversion initially captures a wild-type or deleterious allele with superscript W's and D's. Using their notation, the relevant frequencies at each selected locus in the current generation are p_N, q_N, p_I, and q_I, where q refers to the deleterious allele, p refers to the wild-type allele, and the N and I subscripts denote the Non-inverted and Inverted chromosome classes. Note that these four terms are absolute frequencies, and therefore sum to 1, while $p_I + q_I = I$, the frequency of the inversion. The relative frequency of deleterious alleles on non-inverted and inverted chromosomes are

$$Q_N^W = q_N^W/(1-I) \tag{C1a}$$
$$P_N^W = p_N^W/(1-I) \tag{C1b}$$
$$Q_I^W = q_I^W/I \tag{C1c}$$
$$P_I^W = p_I^W/I. \tag{C1d}$$

The relative frequencies at D loci are calculated in exactly the same way. The frequencies $p^{\cdot}_{\cdot}$ and $q^{\cdot}_{\cdot}$ at each locus change in response to selection and mutation (we continue to ignore backmutation), and the order of events in the life cycle proceeds: mutation → selection → random mating. The frequencies after mutation are

$$q_N^{W,m} = q_N^W + p_N^W \mu \tag{C2a}$$
$$p_N^{W,m} = p_N^W(1-\mu) \tag{C2b}$$
$$q_I^{W,m} = q_I^W + p_I^W \mu \tag{C2c}$$
$$p_I^{W,m} = p_I^W(1-\mu), \tag{C2d}$$

for W loci, and

$$q_N^{D,m} = q_N^D + p_N^D \mu \tag{C3a}$$
$$p_N^{D,m} = p_N^D(1-\mu) \tag{C3b}$$
$$q_I^{D,m} = I \tag{C3c}$$
$$p_I^{D,m} = 0, \tag{C3d}$$

where μ is the mutation rate. The change in inversion frequency is a function of the number of loci spanned by the inversion, the deleterious allele frequencies at those loci, and the selection and dominance coefficients, s and h. The recursions describing the per-generation change in inversion frequency is given by

$$I' = \Big[I^2(1-s)^d\big(1-s(2hQ_I^W P_I^W + Q_I^{W2})\big)^{n-d} + \\ I(1-I)\big(1-s(hP_N^D + Q_N^D)\big)^d\Big(1-s\big(h(Q_N^W P_I^W + P_N^W Q_I^W) + Q_N^W Q_I^W\big)\Big)^{n-d}\Big] \Big/ \overline{w}_I \tag{C4}$$

where $\overline{w}_I$ is equal to

$$\begin{aligned}\overline{w}_I =& I^2\left[(1-s)^d\Big(1-s\big(2hQ_I^W P_I^W+Q_I^{W2}\big)\Big)^{n-d}\right]+\\ & 2I(1-I)\left[\big(1-s(2hP_N^D+Q_N^D)\big)^d\Big(1-s\big(h(Q_N^W P_I^W+P_N^W Q_I^W)+Q_N^W Q_I^W\big)\Big)^{n-d}\right]+\\ & (1-I)^2\left[\big(1-s(2hQ_N^D P_N^D+Q_N^{D2})\big)^d\big(1-s(2hQ_N^W P_N^W+Q_N^{W2})\big)^{n-d}\right]\end{aligned} \tag{C5}$$

The recursions for deleterious allele absolute frequencies are as follows:

$$q_I^{W\prime} = \frac{q_I^{W,m}\Big(1-s\Big(h\big(p_I^{W,m}+p_N^{W,m}\big)+q_I^{W,m}+q_N^{W,m}\Big)\Big)}{I-q_I^{W,m}s\Big(h\big(p_I^{W,m}+p_N^{W,m}\big)+q_I^{W,m}+q_N^{W,m}\Big)}I' \tag{C6a}$$

$$p_I^{W\prime} = I'-q_I^{W\prime} \tag{C6b}$$

$$q_N^{W\prime} = \frac{q_N^{W,m}\Big(1-s\Big(h\big(p_N^{W,m}+p_I^{W,m}\big)+q_N^{W,m}+q_I^{W,m}\Big)\Big)}{1-I-q_N^{W,m}s\Big(h\big(p_N^{W,m}+p_I^{W,m}\big)+q_N^{W,m}+q_I^{W,m}\Big)}(1-I') \tag{C6c}$$

$$p_N^{W\prime} = 1-I'-q_N^{W\prime} \tag{C6d}$$

$$q_N^{D\prime} = \frac{q_N^{D,m}\Big(1-s\big(hp_N^{D,m}+I+q_N^{D,m}\big)\Big)}{1-I-q_N^{D,m}s\big(hp_N^{D,m}+I+q_N^{D,m}\big)}(1-I') \tag{C6e}$$

$$p_N^{D\prime} = 1-I'-q_N^{D\prime} \tag{C6f}$$

Encouragingly, the system of recursions described by Eq(C4 – C6) reproduces the same dynamics as presented in Fig. 1 of Nei et al. (1967) for the special case of completely recessive mutations ($h=0$):

Case	I_0	s	n_{tot}
1	10^-5	0.01	100
2	10^-5	0.01	1000
3	10^-5	0.10	100
4	10^-5	0.10	1000
5	10^-3	0.01	100
6	10^-3	0.01	1000
7	10^-3	0.10	100
8	10^-3	0.10	1000

Eq(C4 – C6) provide deterministic dynamics for Autosomal inversions which can be directly compared against those of SLR-expanding inversions described in Appendix B), and to calculate expected allele and inversion frequencies in Wright-Fisher simulations as described in the main text.

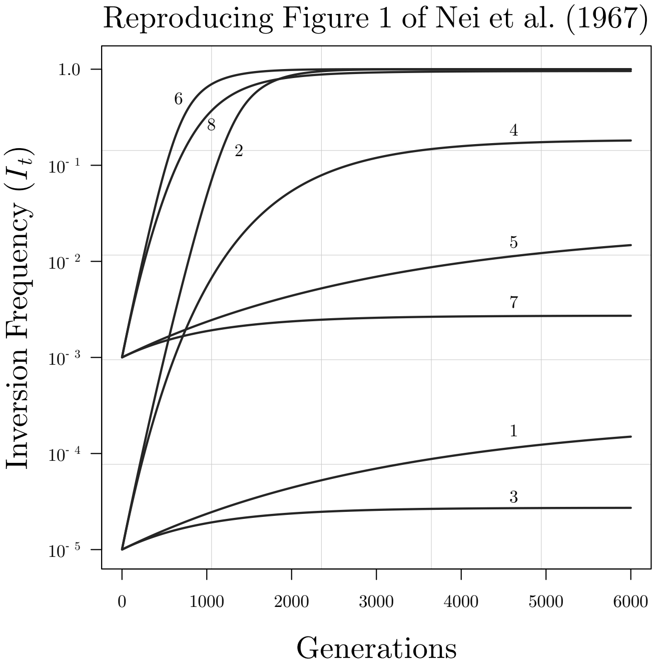

Figure C1: Reproduction of Nei et al. (1967) Figure 1 using the recursions presented in Eq(C4 – C6) to predict deterministic inversion frequency changes over tiem (I). We used the same parameter values as Nei et al. (1967), with $h = 0$, $\mu = 10^{-5}$ and cases 1 – 8 corresponding to:

Appendix D Wright-Fisher simulations

As explained in the main text, our WF simulations make a first pass at studying fixation probabilities of autosomal and SLR-expanding inversions by assuming that deleterious allele frequencies change deterministically due to selection, while the inversion frequency changes in response to both indirect selection and genetic drift. Under the equilbrium assumption used by Connallon and Olito (2020), which we implemented in the results presented in the main text, we can use a significantly simplified model because it is not necessary to track changes in deleterious allele frequencies on any other chromosome class besides the inversion. For autosomal inversions, the time-dependent fitness expressions for each inversion genotype are:

$$w_{SS} = (1-2\mu)^n \tag{D1a}$$

$$w_{SI} = (\hat{q}(1-s) + (1-\hat{q})(1-sh))^d \left(1-u(2-e^{-sht})\right)^{n-d} \tag{D1b}$$

$$w_{II} = (1-s)^d \left(1-2u(1-e^{-sht})\right)^{n-d}, \tag{D1c}$$

where S and I subscripts refer to standard and inverted chromosomes, respectively. These fitness expressions were

then used in the following autosomal genotypic frequency recursions:

$$F_{SS,t+1} = p_t^2 w_{SS}/\overline{w} \tag{D2a}$$

$$F_{SI,t+1} = 2p_t q_t w_{IS,t}/\overline{w} \tag{D2b}$$

$$F_{II,t+1} = q_t^2 w_{II,t}/\overline{w}, \tag{D2c}$$

where

$$\overline{w} = p_t^2 w_{SS} + 2p_t q_t w_{IS,t} + q_t^2 w_{II,t}, \tag{D3}$$

where $p = F_{SS} + F_{SI}/2$ and $q = F_{SI}/2 + F_{II}$ are the frequencies of standard and inverted haplotypes, respectively. As described in the main text, the genotypic recursions given in Eq(D2a) were used to predict the expected adult genotypic frequencies in generation $t+1$, and we performed WF simulations using multinomial pseudo-random sampling of N adult genotypes to generate the realized frequencies (e.g., Charlesworth and Charlesworth 2010, pp. 229-230).

For SLR-expanding inversions, the corresponding fitness expressions for males under the equilibrium approximation are simply:

$$w_{XY} = (1-2\mu)^n \tag{D4a}$$

$$w_{XY_t^I} = (\hat{q}(1-s) + (1-\hat{q})(1-sh))^d \left(1 - u(2-e^{-sht})\right)^{n-d}, \tag{D4b}$$

which are the same as the autosomal expressions for w_{SS} and w_{SI}. The expressions also underscore the main intuition from both our deterministic results and the WF models using an equilibrium approximation: the models can only diverge under conditions where autosomal inversion-homozygotes occur with reasonably high frequency. For this to happen, deleterious alleles must be segregating at high equilibrium frequencies, which requires highly recessive mutations and/or weak selection (sh is small), as well as high mutation rates (large U). The above fitness expressions were used in a standard haploid recursion for the frequency of inverted Y chromosomes:

$$Y_{t+1}^I = Y_t^I w_{XY_t^I}/\overline{w}_Y \tag{D5}$$

where

$$\overline{w}_Y = Y_t^I w_{XY_I} + Y_t w_{XY}. \tag{D6}$$

As described in the main text, the deterministic recursion given by Eq(D5) was used to predict the expected inversion frequency in generation $t+1$, which served as the binomial probability of sampling an inverted Y haplotype for $N/2$ Y chromosomes, which were used to generate the realized inversion frequency in the WF simulations.

Appendix E Supplementary Figures

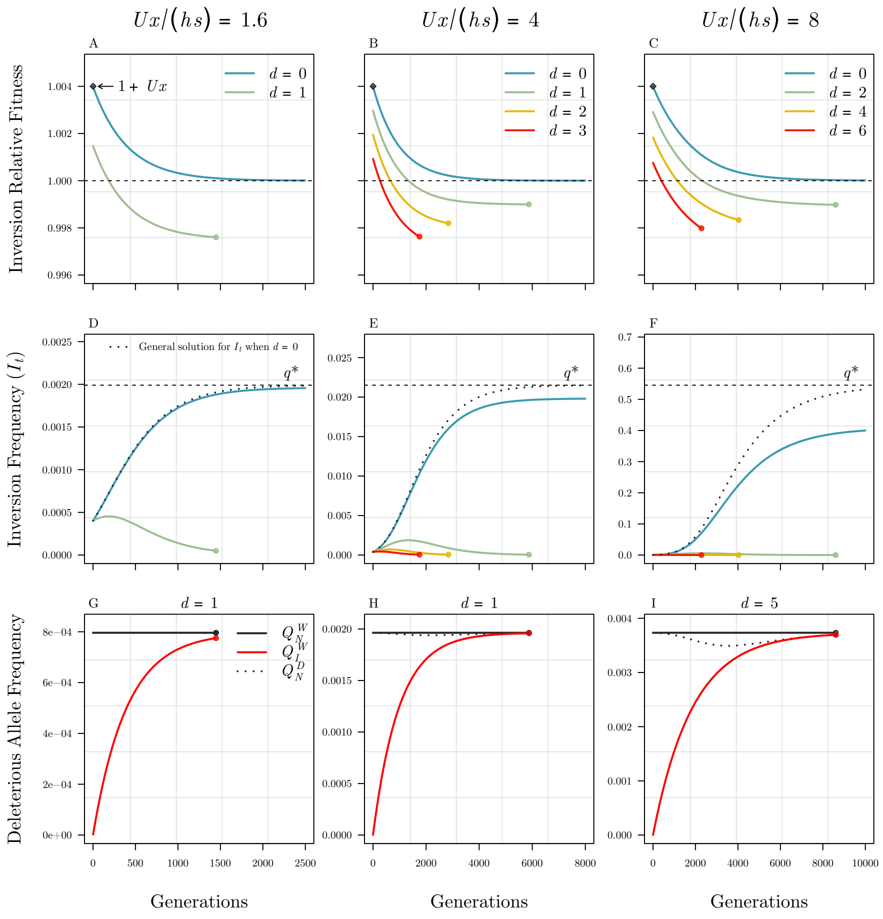

Figure S1: Illustration of deterministic fitness and frequency dynamics for **autosomal inversions** initially loaded with different numbers of deleterious alleles. Results are shown for the same parameter values as Fig. 1 in the main text: inversions of length $x = 0.2$, and three different dominance scenarios ($h = \{0.25, 0.1, 0.05\}$, yeilding average deleterious mutation loads over the chromosomal segment spanned by inversions of $Ux/(hs) = \{1.6, 4, 40\}$, corresponding to each column of panels, left to right). Panels (A-C) show the fitness of SLR-expanding inversions on a Y chromosome relative to the average fitness of all Y chromosomes (color coded lines). Colored points indicate when the corresponding inversions dropped below a frequency of 10^{-5}, where they became effectively extinct, while black stars indicate when an inversion reached a frequency of $(1-10^{-5})$, at which point they were considered to have fixed. Black diamonds indicate the analytic approximation for initial relative fitness of unloaded inversions ($d = 0$) in the first generation. Panels (D-F) show the inversion frequency dynamics (color coded lines) and illustrate that despite being initially beneficial, lightly loaded inversions (*i*) are not expected to deterministically rise to high frequencies unless deleterious mutations are strongly recessive, selection is very weak, or both (i.e., *sh* is small) ; and (*ii*) will eventually become deleterious and crash to extinction (see Supplementary Figures in Appendix D). The horizontal dashed lines benchmark the asymptotic frequency of initially unloaded inversions, q^*, while the dotted line shows the corresponding discrete-time general solution for $Y_{I,t}$ from which q^* is derived. Panels (G-I) illustrate the deleterious allele frequency dynamics at W loci on the inversion (Q_I^W; red line), and both W and D loci on non-inverted chromosomes (Q_N^W and Q_N^D; black solid and dashed lines respectively) for the representative case of inversions initially loaded with relatively few deleterious alleles ($d = 1$ for G,H; $d = 5$ for I). Results were generated using the following parameter values: $s = 0.01$, $U = 0.02$, $x = 0.2$, $n_{tot} = 10^4$.

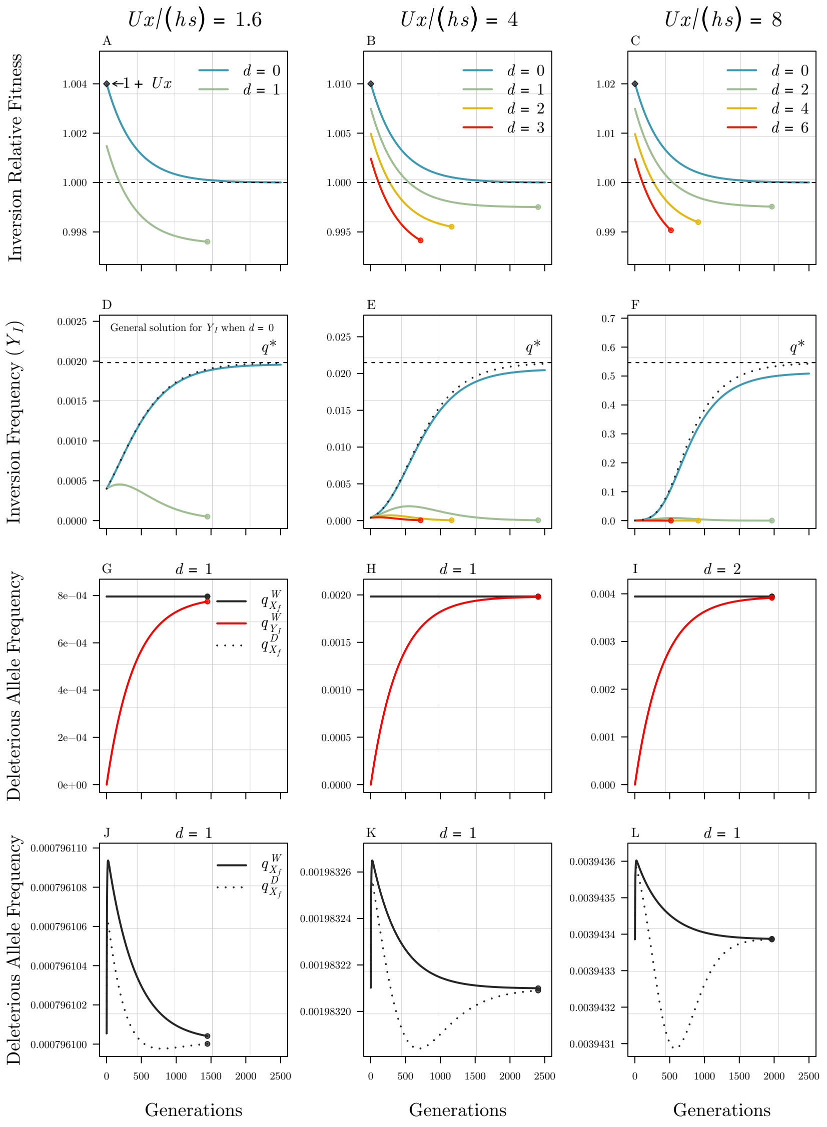

Figure S2: Illustration of deterministic fitness and frequency dynamics for **SLR-expanding inversions** initially loaded with different numbers of deleterious alleles. Results are shown for inversions of length $x = 0.2$, and **three different mutation rates** ($U = \{0.02, 0.05, 0.1\}$, yeilding average deleterious mutation loads over the chromosomal segment spanned by inversions of $Ux/(hs) = \{1.6, 4, 8\}$, corresponding to each column of panels, left to right). Panels (A-C) show the fitness of SLR-expanding inversions on a Y chromosome relative to the average fitness of all Y chromosomes (color coded lines). Colored points indicate when the corresponding inversions dropped below a frequency of 10^{-5}, where they became effectively extinct, while black stars indicate when an inversion reached a frequency of $(1-10^{-5})$, at which point they were considered to have fixed. Black diamonds indicate the analytic approximation for initial relative fitness of unloaded inversions ($d = 0$) in the first generation. Panels (D-F) show the inversion frequency dynamics (color coded lines) and illustrate that despite being initially beneficial, lightly loaded inversions (*i*) are not expected to deterministically rise to high frequencies unless deleterious mutations are strongly recessive, selection is very weak, or both (i.e., sh is small) ; and (*ii*) will eventually become deleterious and crash to extinction (see Supplementary figures in Appendix D). The horizontal dashed lines benchmark the asymptotic frequency of initially unloaded inversions, q^*, while the dotted line shows the corresponding discrete-time general solution for $Y_{I,t}$ from which q^* is derived. Panels (G-I) illustrate the deleterious allele frequency dynamics at W loci on the inversion ($q^W_{Y_I}$; red line), and both W and D loci on X chromosomes in ovules/eggs ($q^W_{X_f}$ and $q^D_{X_f}$; black solid and dashed lines respectively) for the representative case of inversions initially loaded with relatively few deleterious alleles ($d = 1$ for G,H; $d = 5$ for I). Results were generated using the following parameter values: $s = 0.01$, $U = 0.02$, $x = 0.2$, $n_{tot} = 10^4$.

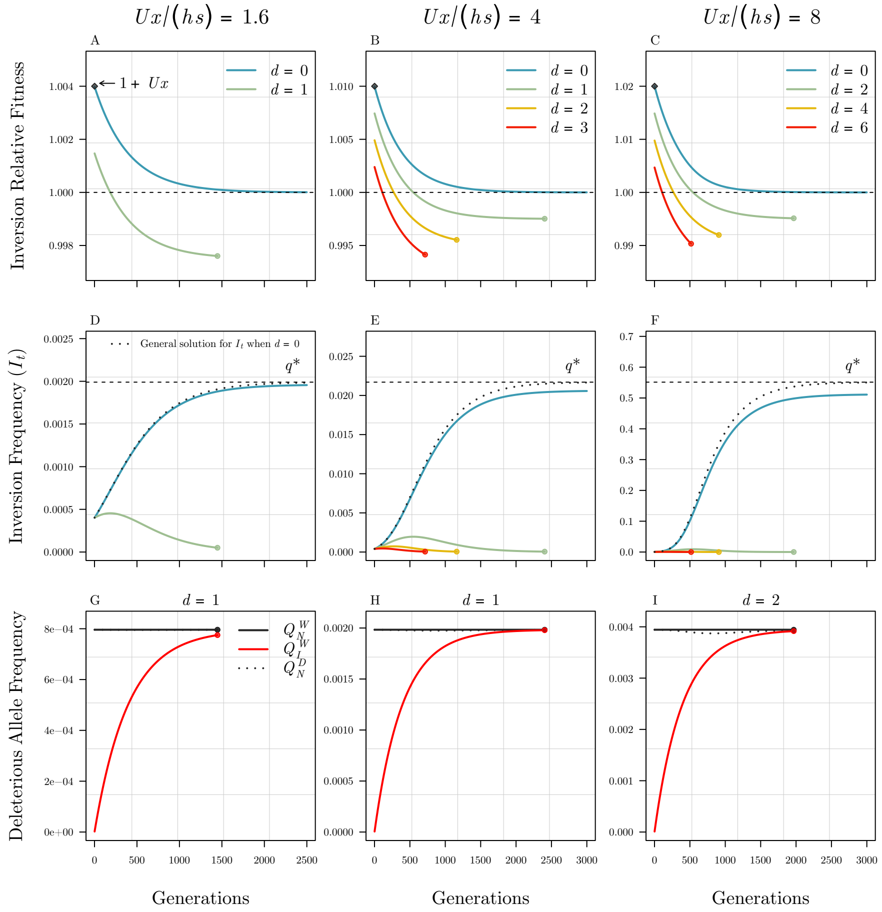

Figure S3: Illustration of deterministic fitness and frequency dynamics for **autosomal inversions** initially loaded with different numbers of deleterious alleles. Results are shown for inversions of length $x = 0.2$, and **three different mutation rates** ($U = \{0.02, 0.05, 0.1\}$, yeilding average deleterious mutation loads over the chromosomal segment spanned by inversions of $Ux/(hs) = \{1.6, 4, 8\}$, corresponding to each column of panels, left to right). Panels (A-C) show the fitness of SLR-expanding inversions on a Y chromosome relative to the average fitness of all Y chromosomes (color coded lines). Colored points indicate when the corresponding inversions dropped below a frequency of 10^{-5}, where they became effectively extinct, while black stars indicate when an inversion reached a frequency of $(1-10^{-5})$, at which point they were considered to have fixed. Black diamonds indicate the analytic approximation for initial relative fitness of unloaded inversions ($d = 0$) in the first generation. Panels (D-F) show the inversion frequency dynamics (color coded lines) and illustrate that despite being initially beneficial, lightly loaded inversions (i) are not expected to deterministically rise to high frequencies unless deleterious mutations are strongly recessive, selection is very weak, or both (i.e., sh is small) ; and (ii) will eventually become deleterious and crash to extinction (see Supplementary figures in Appendix D). The horizontal dashed lines benchmark the asymptotic frequency of initially unloaded inversions, q^*, while the dotted line shows the corresponding discrete-time general solution for $Y_{I,t}$ from which q^* is derived. Panels (G-I) illustrate the deleterious allele frequency dynamics at W loci on the inversion (Q_I^W; red line), and both W and D loci on X chromosomes in ovules/eggs (Q_N^W and Q_N^D; black solid and dashed lines respectively) for the representative case of inversions initially loaded with relatively few deleterious alleles ($d = 1$ for G,H; $d = 5$ for I). Results were generated using the following parameter values: $s = 0.01$, $U = 0.02$, $x = 0.2$, $n_{tot} = 10^4$.

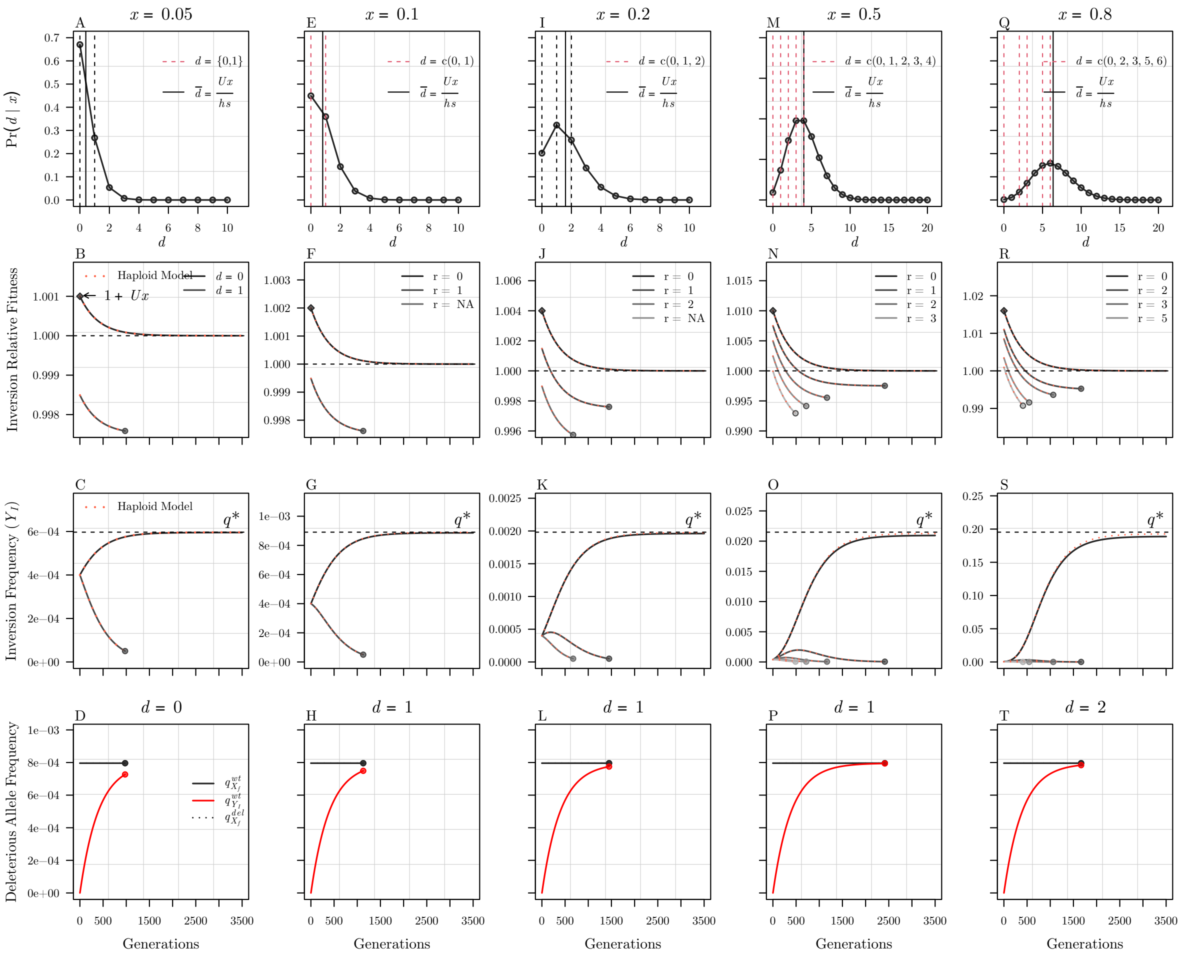

Figure S4: Overview of deterministic fitness and frequency dynamics for initially beneficial **SLR-expanding inversions** of different sizes initially loaded with different numbers of **partially recessive** deleterious alleles with a **low mutation rate** ($h = 0.25$ and $U = 0.02$; *compare with Fig.* S10). Each column of panels presents results for inversions of lengths $x = 0.05$ (A–D), 0.1 (E–H), 0.2 (I–L), 0.5 (M–P), and 0.8 (Q–T). The first row of panels (A,E,I,M,Q) shows the probability that an inversion of length x captures d deleterious alleles (points and black lines), with benchmarks (vertical red dashed lines) showing the values of d being illustrated in the corresponding column of panels. Values of d were chosen to (more or less) evenly cover the lower half of the distribution of $\Pr(d|x)$. As in Fig. 1 of the main text, the lower three rows of panels illustrate changes in inversion relative fitness (2nd row), inversion frequency (3rd row), and deleterious allele frequencies at *wt* loci on the inversion ($q^W_{Y_I}$; red line), and both *wt* and *del* loci on X chromosomes in ovules/eggs ($q^W_{X_f}$ and $q^D_{X_f}$; black solid and dashed lines respectively) (4th row). Results were generated using the following parameter values: $h = 0.25$, $s = 0.01$, $U = 0.02$, $n_{tot} = 10^4$.

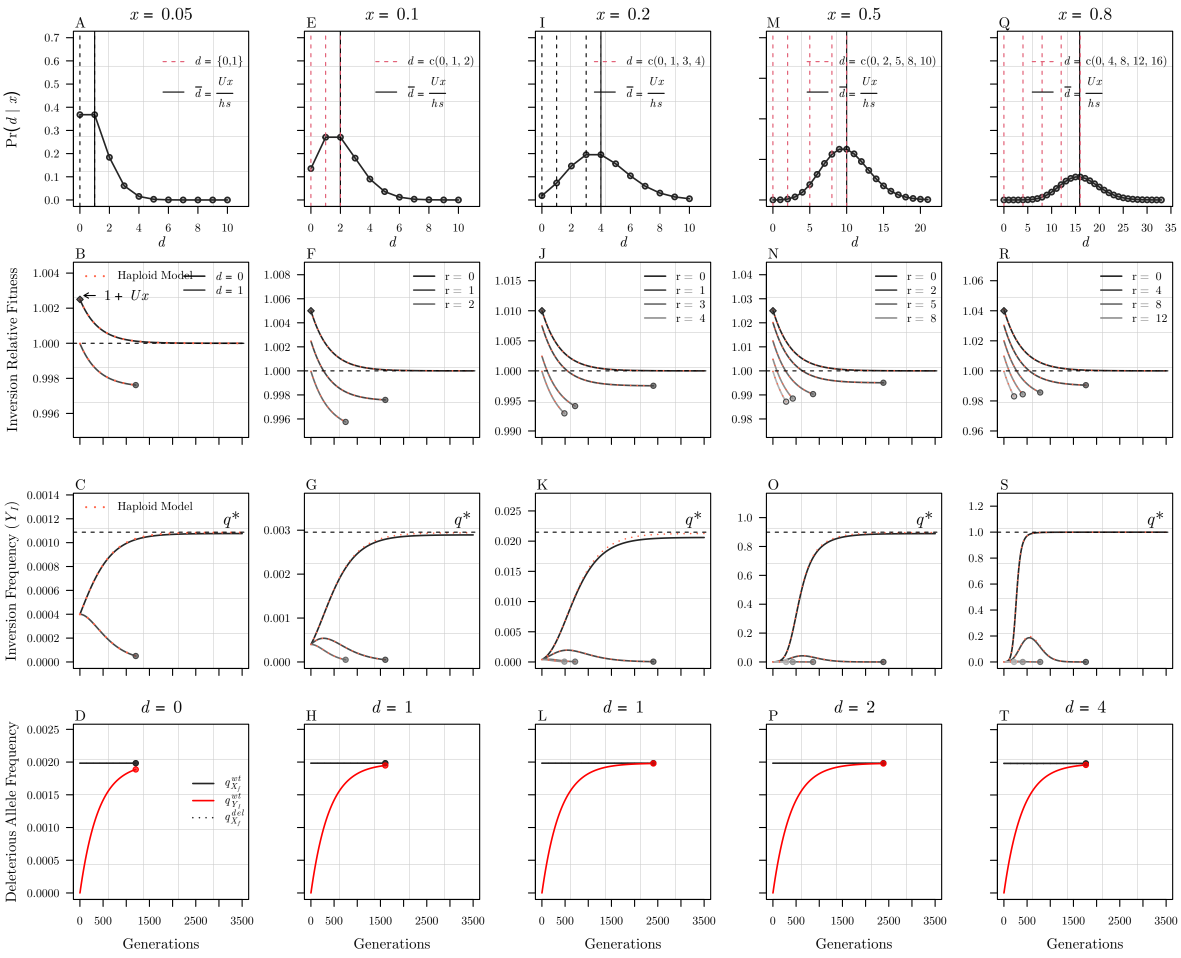

Figure S5: Overview of deterministic fitness and frequency dynamics for initially beneficial **SLR-expanding inversions** of different sizes initially loaded with different numbers of **partially recessive** deleterious alleles with an **intermediate mutation rate** ($h = 0.25$ and $U = 0.05$; *compare with Fig.* S11). Each column of panels presents results for inversions of lengths $x = 0.05$ (A–D), 0.1 (E–H), 0.2 (I–L), 0.5 (M–P), and 0.8 (Q–T). The first row of panels (A,E,I,M,Q) shows the probability that an inversion of length x captures d deleterious alleles (points and black lines), with benchmarks (vertical red dashed lines) showing the values of d being illustrated in the corresponding column of panels. Values of d were chosen to (more or less) evenly cover the lower half of the distribution of $\Pr(d|x)$. As in Fig. 1 of the main text, the lower three rows of panels illustrate changes in inversion relative fitness (2nd row), inversion frequency (3rd row), and deleterious allele frequencies at *wt* loci on the inversion ($q^W_{Y_I}$; red line), and both *wt* and *del* loci on X chromosomes in ovules/eggs ($q^W_{X_f}$ and $q^D_{X_f}$; black solid and dashed lines respectively) (4th row). Results were generated using the following parameter values: $h = 0.25$, $s = 0.01$, $U = 0.05$, $n_{tot} = 10^4$.

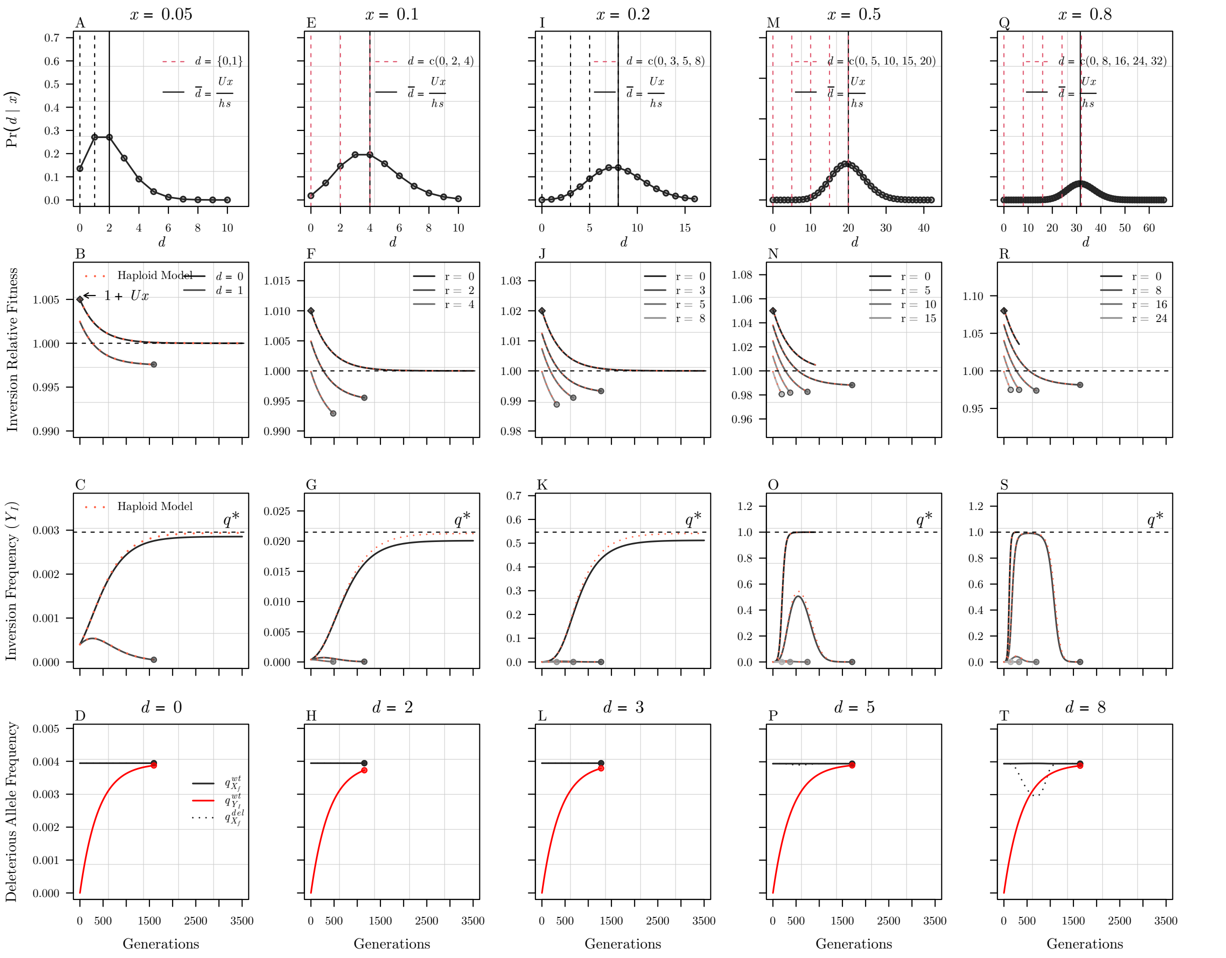

Figure S6: Overview of deterministic fitness and frequency dynamics for initially beneficial **SLR-expanding inversions** of different sizes initially loaded with different numbers of **partially recessive** deleterious alleles with a **high mutation rate** ($h = 0.25$ and $U = 0.1$; *compare with Fig.* S12). Each column of panels presents results for inversions of lengths $x = 0.05$ (A–D), 0.1 (E–H), 0.2 (I–L), 0.5 (M–P), and 0.8 (Q–T). The first row of panels (A,E,I,M,Q) shows the probability that an inversion of length x captures d deleterious alleles (points and black lines), with benchmarks (vertical red dashed lines) showing the values of d being illustrated in the corresponding column of panels. Values of d were chosen to (more or less) evenly cover the lower half of the distribution of $\Pr(d|x)$. As in Fig. 1 of the main text, the lower three rows of panels illustrate changes in inversion relative fitness (2nd row), inversion frequency (3rd row), and deleterious allele frequencies at *wt* loci on the inversion ($q^W_{Y_I}$; red line), and both *wt* and *del* loci on X chromosomes in ovules/eggs ($q^W_{X_f}$ and $q^D_{X_f}$; black solid and dashed lines respectively) (4th row). Results were generated using the following parameter values: $h = 0.25$, $s = 0.01$, $U = 0.1$, $n_{tot} = 10^4$.

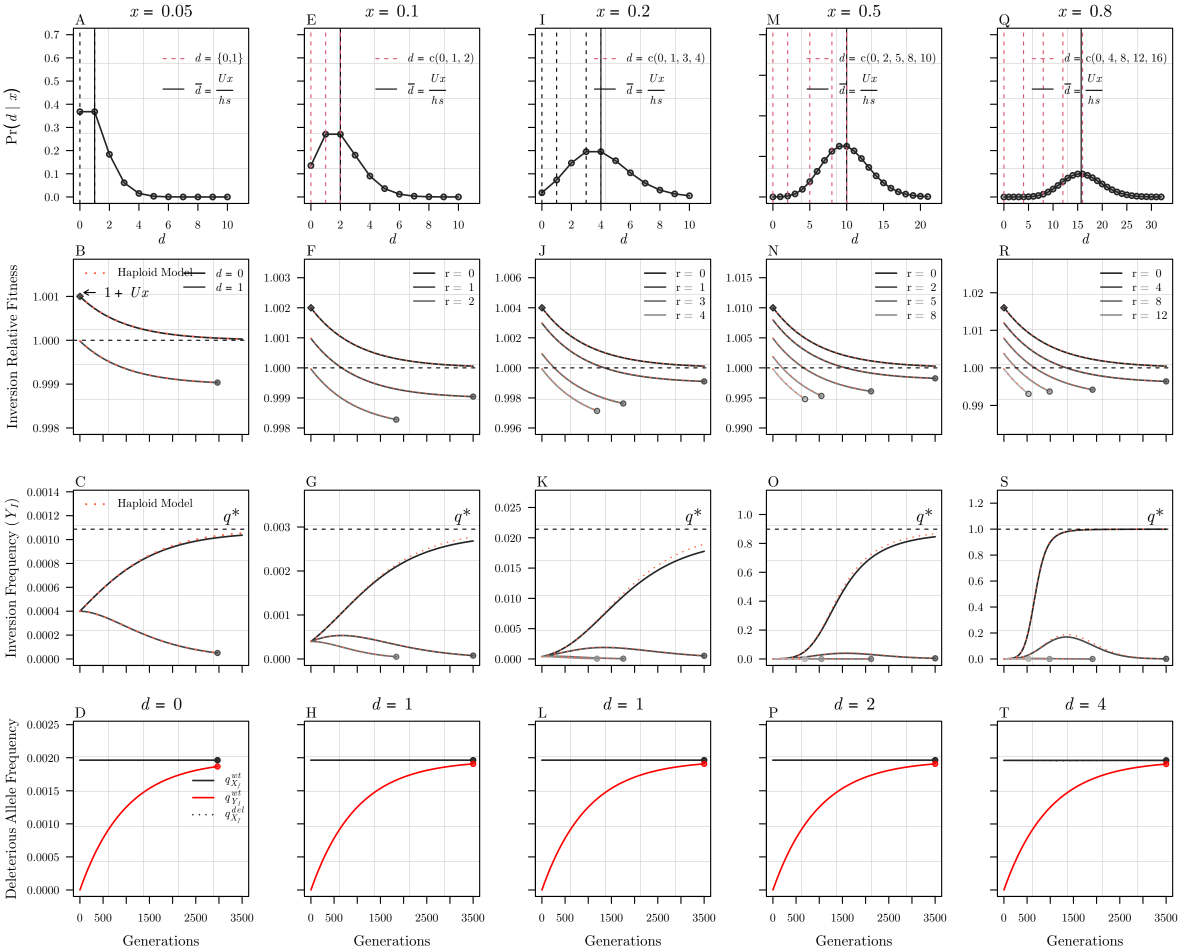

Figure S7: Overview of deterministic fitness and frequency dynamics for initially beneficial **SLR-expanding inversions** of different sizes initially loaded with different numbers of **strongly recessive** deleterious alleles with a **low mutation rate** ($h = 0.1$ and $U = 0.02$; *compare with Fig.* S13) deleterious alleles. Each column of panels presents results for inversions of lengths $x = 0.05$ (A–D), 0.1 (E–H), 0.2 (I–L), 0.5 (M–P), and 0.8 (Q–T). The first row of panels (A,E,I,M,Q) shows the probability that an inversion of length x captures d deleterious alleles (points and black lines), with benchmarks (vertical red dashed lines) showing the values of d being illustrated in the corresponding column of panels. Values of d were chosen to (more or less) evenly cover the lower half of the distribution of $\Pr(d|x)$. As in Fig. 1 of the main text, the lower three rows of panels illustrate changes in inversion relative fitness (2nd row), inversion frequency (3rd row), and deleterious allele frequencies at *wt* loci on the inversion ($q^W_{Y_I}$; red line), and both *wt* and *del* loci on X chromosomes in ovules/eggs ($q^W_{X_f}$ and $q^D_{X_f}$; black solid and dashed lines respectively) (4th row). Results were generated using the following parameter values: $h = 0.1$, $s = 0.01$, $U = 0.02$, $n_{tot} = 10^4$.

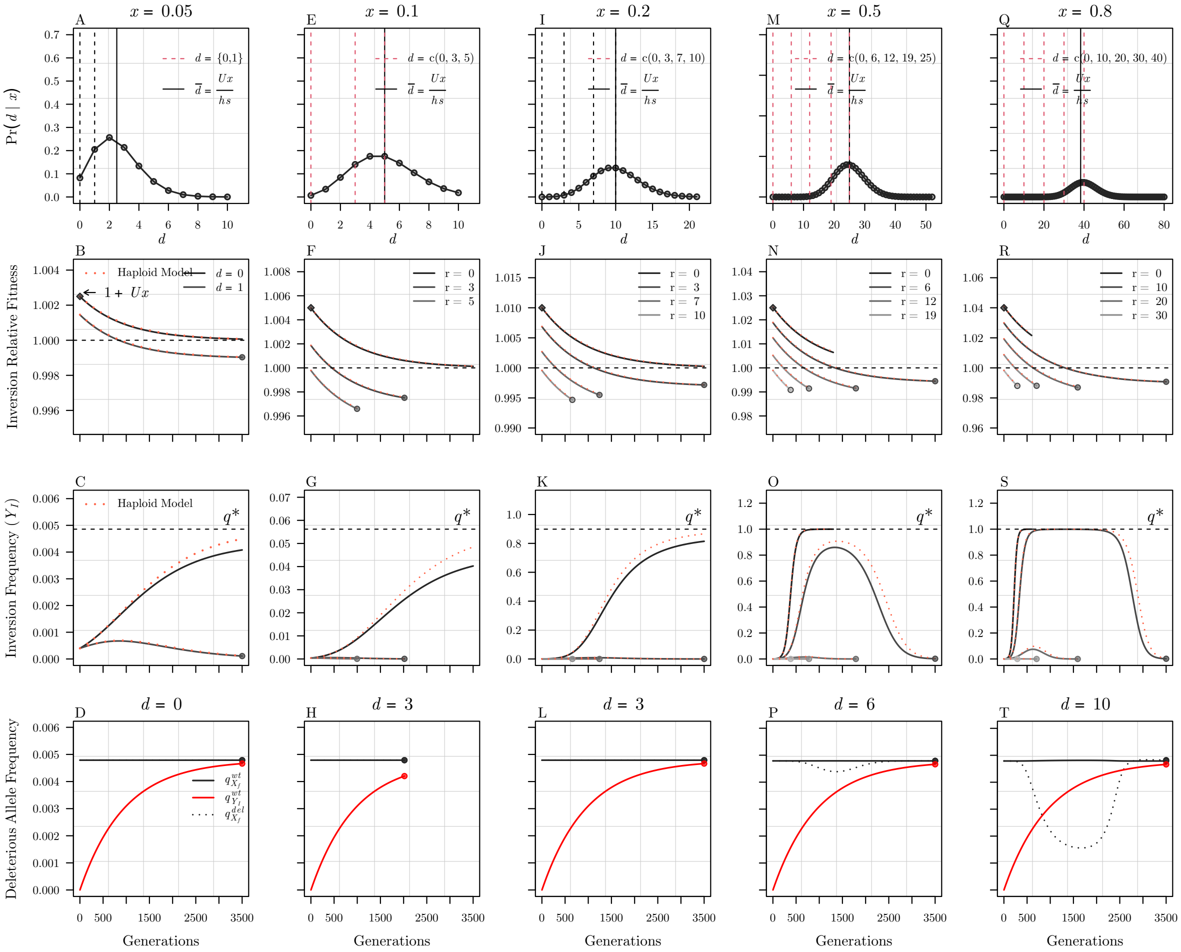

Figure S8: Overview of deterministic fitness and frequency dynamics for initially beneficial **SLR-expanding inversions** of different sizes initially loaded with different numbers of **strongly recessive** deleterious alleles with an **intermediate mutation rate** ($h = 0.1$ and $U = 0.05$; *compare with Fig.* S14). Each column of panels presents results for inversions of lengths $x = 0.05$ (A–D), 0.1 (E–H), 0.2 (I–L), 0.5 (M–P), and 0.8 (Q–T). The first row of panels (A,E,I,M,Q) shows the probability that an inversion of length x captures d deleterious alleles (points and black lines), with benchmarks (vertical red dashed lines) showing the values of d being illustrated in the corresponding column of panels. Values of d were chosen to (more or less) evenly cover the lower half of the distribution of $\Pr(d|x)$. As in Fig. 1 of the main text, the lower three rows of panels illustrate changes in inversion relative fitness (2nd row), inversion frequency (3rd row), and deleterious allele frequencies at *wt* loci on the inversion ($q^{W}_{Y_I}$; red line), and both *wt* and *del* loci on X chromosomes in ovules/eggs ($q^{W}_{X_f}$ and $q^{D}_{X_f}$; black solid and dashed lines respectively) (4th row). Results were generated using the following parameter values: $h = 0.1$, $s = 0.01$, $U = 0.05$, $n_{tot} = 10^4$.

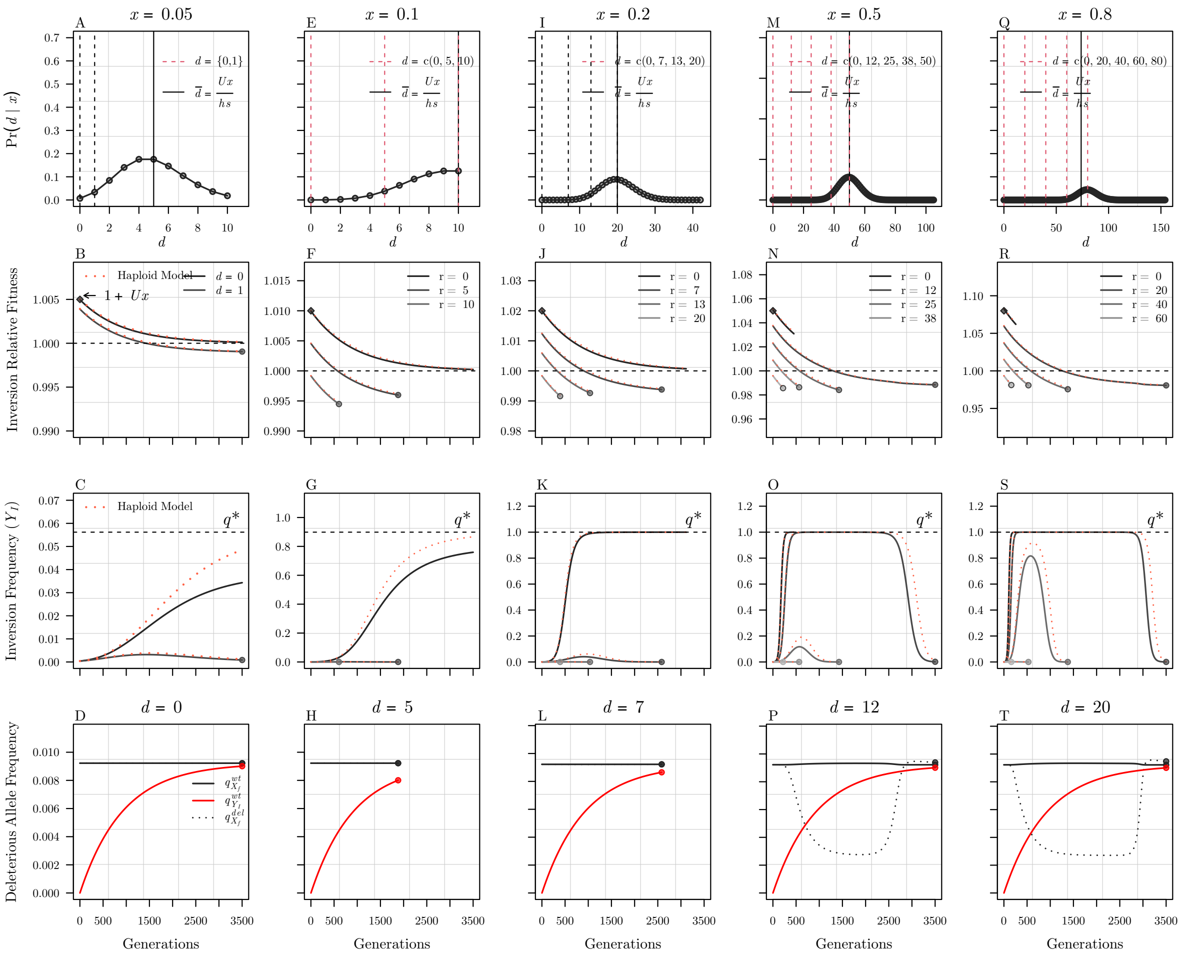

Figure S9: Overview of deterministic fitness and frequency dynamics for initially beneficial **SLR-expanding inversions** of different sizes initially loaded with different numbers of **strongly recessive** deleterious alleles with a **high mutation rate** ($h = 0.1$ and $U = 0.1$; *compare with Fig.* S15). Each column of panels presents results for inversions of lengths $x = 0.05$ (A–D), 0.1 (E–H), 0.2 (I–L), 0.5 (M–P), and 0.8 (Q–T). The first row of panels (A,E,I,M,Q) shows the probability that an inversion of length x captures d deleterious alleles (points and black lines), with benchmarks (vertical red dashed lines) showing the values of d being illustrated in the corresponding column of panels. Values of d were chosen to (more or less) evenly cover the lower half of the distribution of $\Pr(d|x)$. As in Fig. 1 of the main text, the lower three rows of panels illustrate changes in inversion relative fitness (2nd row), inversion frequency (3rd row), and deleterious allele frequencies at *wt* loci on the inversion ($q^W_{Y_I}$; red line), and both *wt* and *del* loci on X chromosomes in ovules/eggs ($q^W_{X_f}$ and $q^D_{X_f}$; black solid and dashed lines respectively) (4th row). Results were generated using the following parameter values: $h = 0.1$, $s = 0.01$, $U = 0.12$, $n_{tot} = 10^4$.

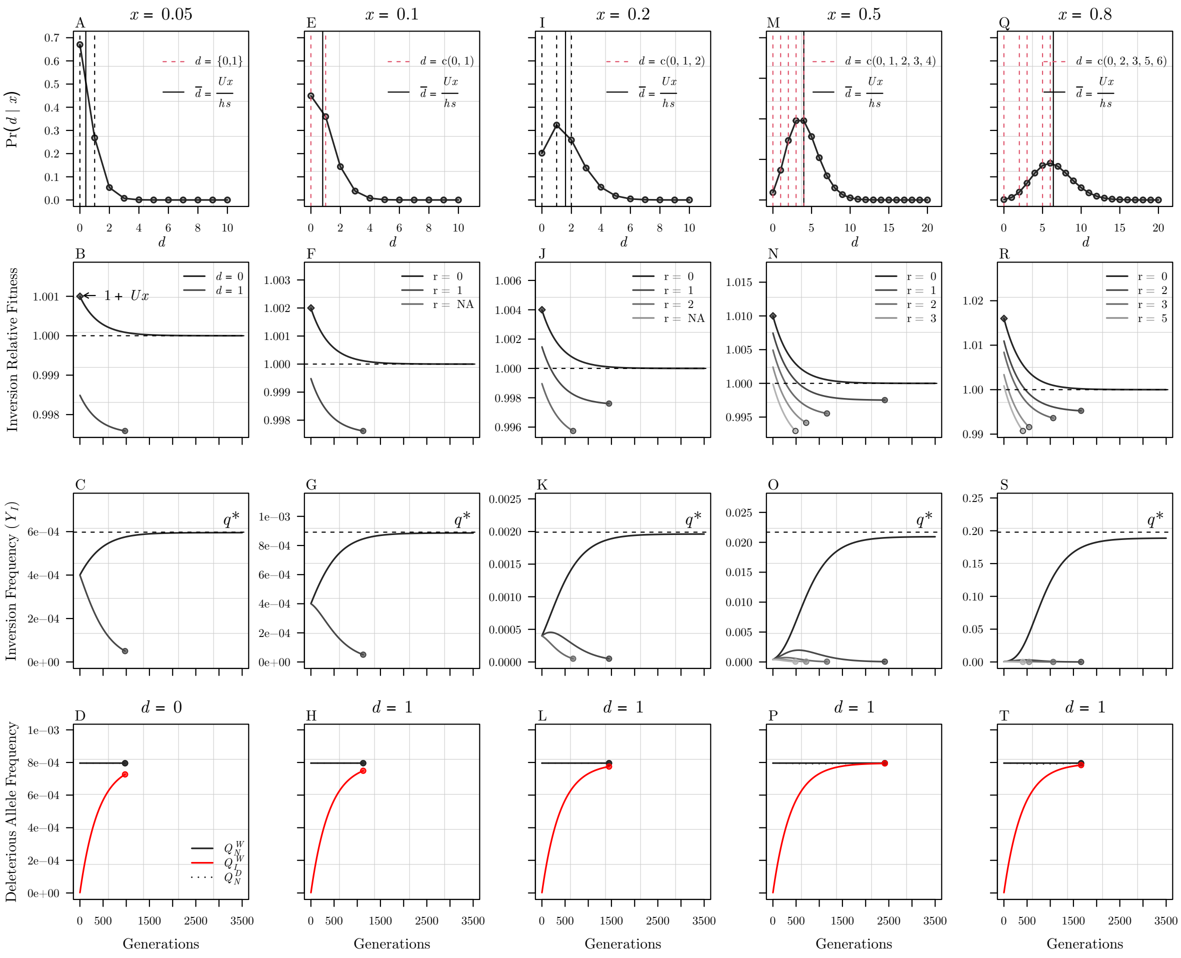

Figure S10: Overview of deterministic fitness and frequency dynamics for initially beneficial **Autosomal inversions** of different sizes initially loaded with different numbers of **partially recessive** deleterious alleles with a **low mutation rate** ($h = 0.25$ and $U = 0.02$) deleterious alleles. Each column of panels presents results for inversions of lengths $x =$ 0.05 (A–D), 0.1 (E–H), 0.2 (I–L), 0.5 (M–P), and 0.8 (Q–T). The first row of panels (A,E,I,M,Q) shows the probability that an inversion of length x captures r deleterious alleles (points and black lines), with benchmarks (vertical red dashed lines) showing the values of r being illustrated in the corresponding column of panels. Values of d were chosen to (more or less) evenly cover the lower half of the distribution of $\Pr(d|x)$. As in Fig. 1 of the main text, the lower three rows of panels illustrate changes in inversion relative fitness (2nd row), inversion frequency (3rd row), and deleterious allele frequencies at *wt* loci on the inversion ($q_{Y_I}^W$; red line), and both *wt* and *del* loci on X chromosomes in ovules/eggs ($q_{X_f}^W$ and $q_{X_f}^D$; black solid and dashed lines respectively) (4th row). Results were generated using the following parameter values: $h = 0.25$, $s = 0.01$, $U = 0.02$, $n_{tot} = 10^4$.

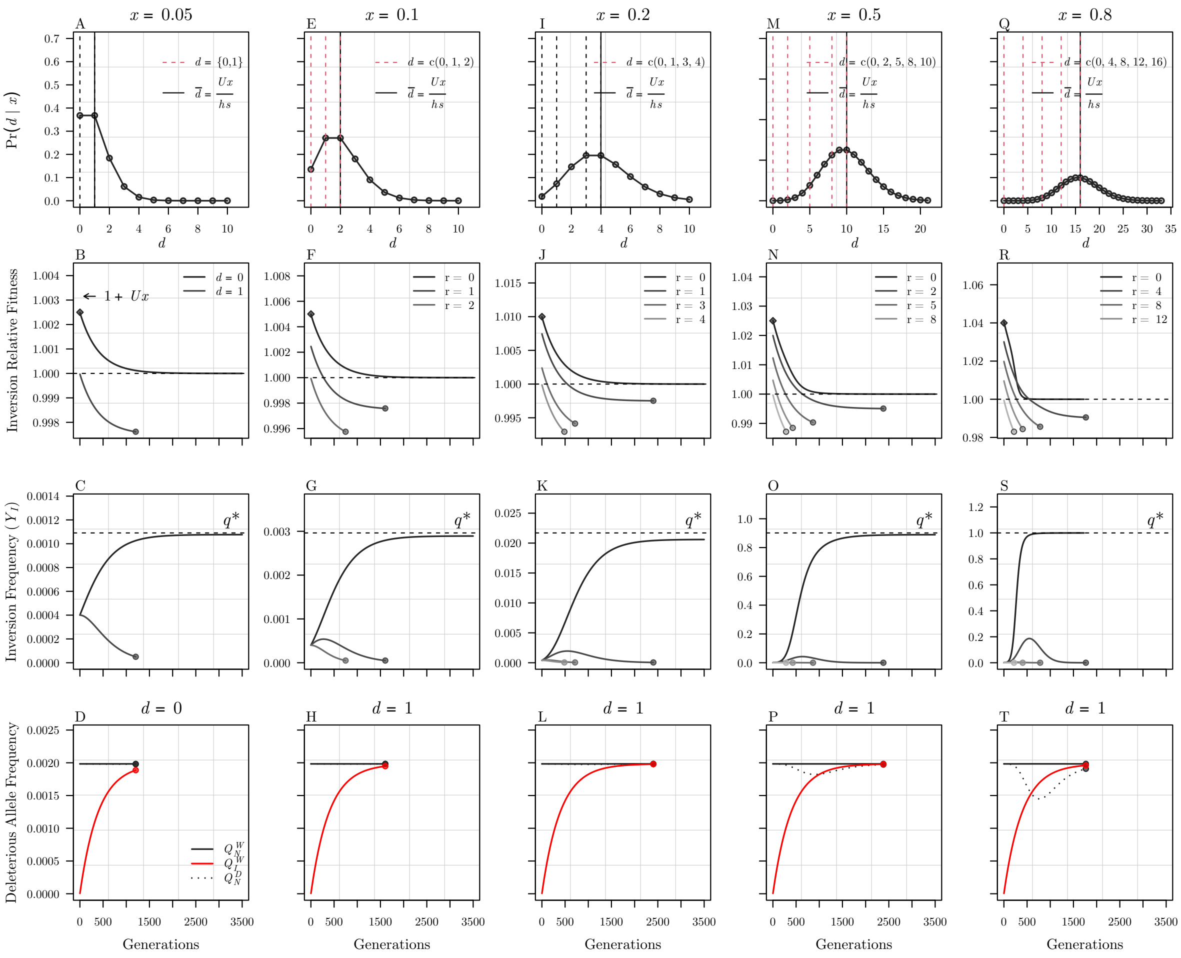

Figure S11: Overview of deterministic fitness and frequency dynamics for initially beneficial **Autosomal inversions** of different sizes initially loaded with different numbers of **partially recessive** deleterious alleles with an **intermediate mutation rate** ($h = 0.25$ and $U = 0.05$). Each column of panels presents results for inversions of lengths $x = 0.05$ (A–D), 0.1 (E–H), 0.2 (I–L), 0.5 (M–P), and 0.8 (Q–T). The first row of panels (A,E,I,M,Q) shows the probability that an inversion of length x captures d deleterious alleles (points and black lines), with benchmarks (vertical red dashed lines) showing the values of d being illustrated in the corresponding column of panels. Values of d were chosen to (more or less) evenly cover the lower half of the distribution of $\Pr(d|x)$. As in Fig. 1 of the main text, the lower three rows of panels illustrate changes in inversion relative fitness (2nd row), inversion frequency (3rd row), and deleterious allele frequencies at *wt* loci on the inversion ($q^W_{Y_I}$; red line), and both *wt* and *del* loci on X chromosomes in ovules/eggs ($q^W_{X_f}$ and $q^D_{X_f}$; black solid and dashed lines respectively) (4th row). Results were generated using the following parameter values: $h = 0.25$, $s = 0.01$, $U = 0.05$, $n_{tot} = 10^4$.

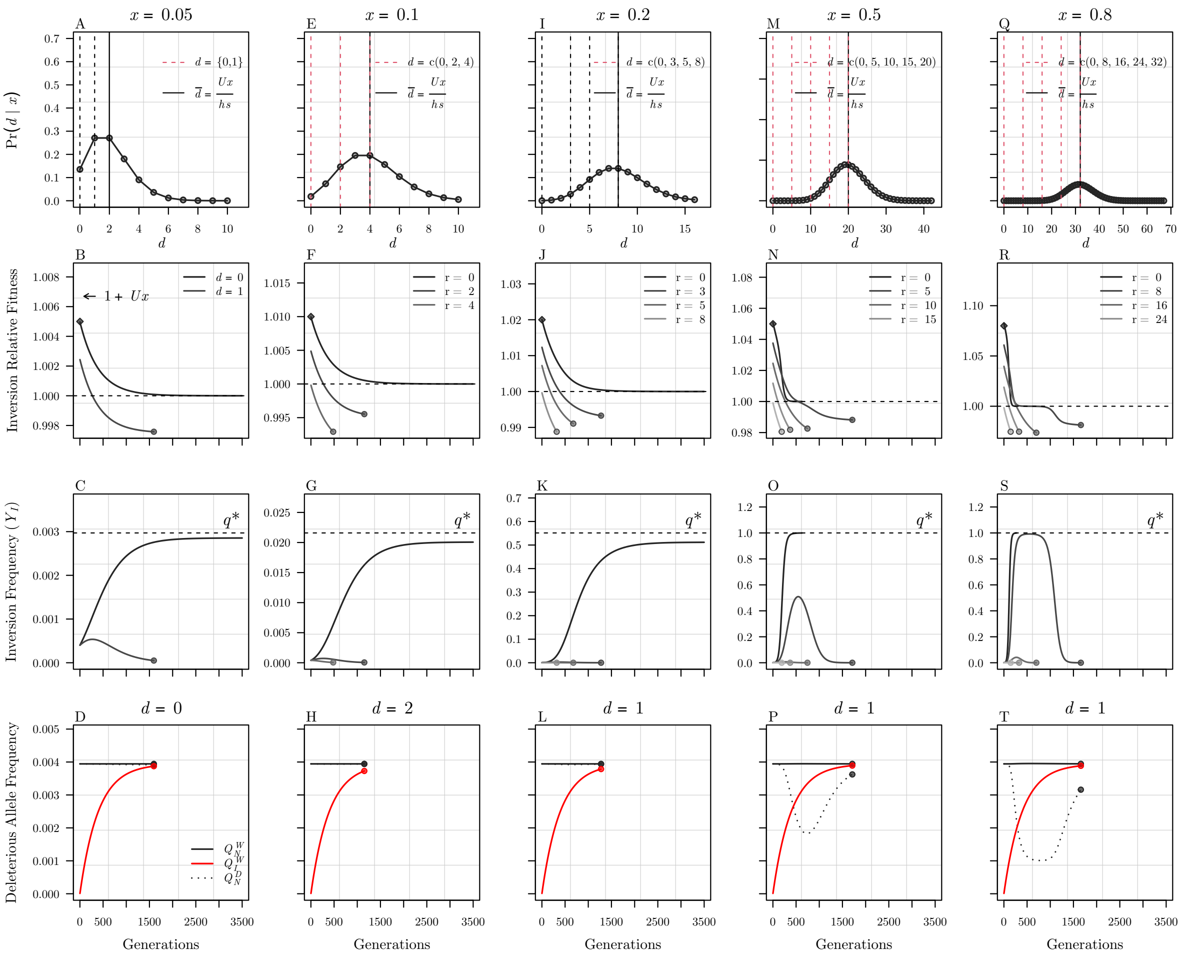

Figure S12: Overview of deterministic fitness and frequency dynamics for initially beneficial **Autosomal inversions** of different sizes initially loaded with different numbers of **partially recessive** deleterious alleles with a **high mutation rate** ($h = 0.25$ and $U = 0.05$). Each column of panels presents results for inversions of lengths $x = 0.05$ (A–D), 0.1 (E–H), 0.2 (I–L), 0.5 (M–P), and 0.8 (Q–T). The first row of panels (A,E,I,M,Q) shows the probability that an inversion of length x captures d deleterious alleles (points and black lines), with benchmarks (vertical red dashed lines) showing the values of d being illustrated in the corresponding column of panels. Values of d were chosen to (more or less) evenly cover the lower half of the distribution of $\Pr(d|x)$. As in Fig. 1 of the main text, the lower three rows of panels illustrate changes in inversion relative fitness (2nd row), inversion frequency (3rd row), and deleterious allele frequencies at *wt* loci on the inversion ($q^W_{Y_I}$; red line), and both *wt* and *del* loci on X chromosomes in ovules/eggs ($q^W_{X_f}$ and $q^D_{X_f}$; black solid and dashed lines respectively) (4th row). Results were generated using the following parameter values: $h = 0.25$, $s = 0.01$, $U = 0.1$, $n_{tot} = 10^4$.

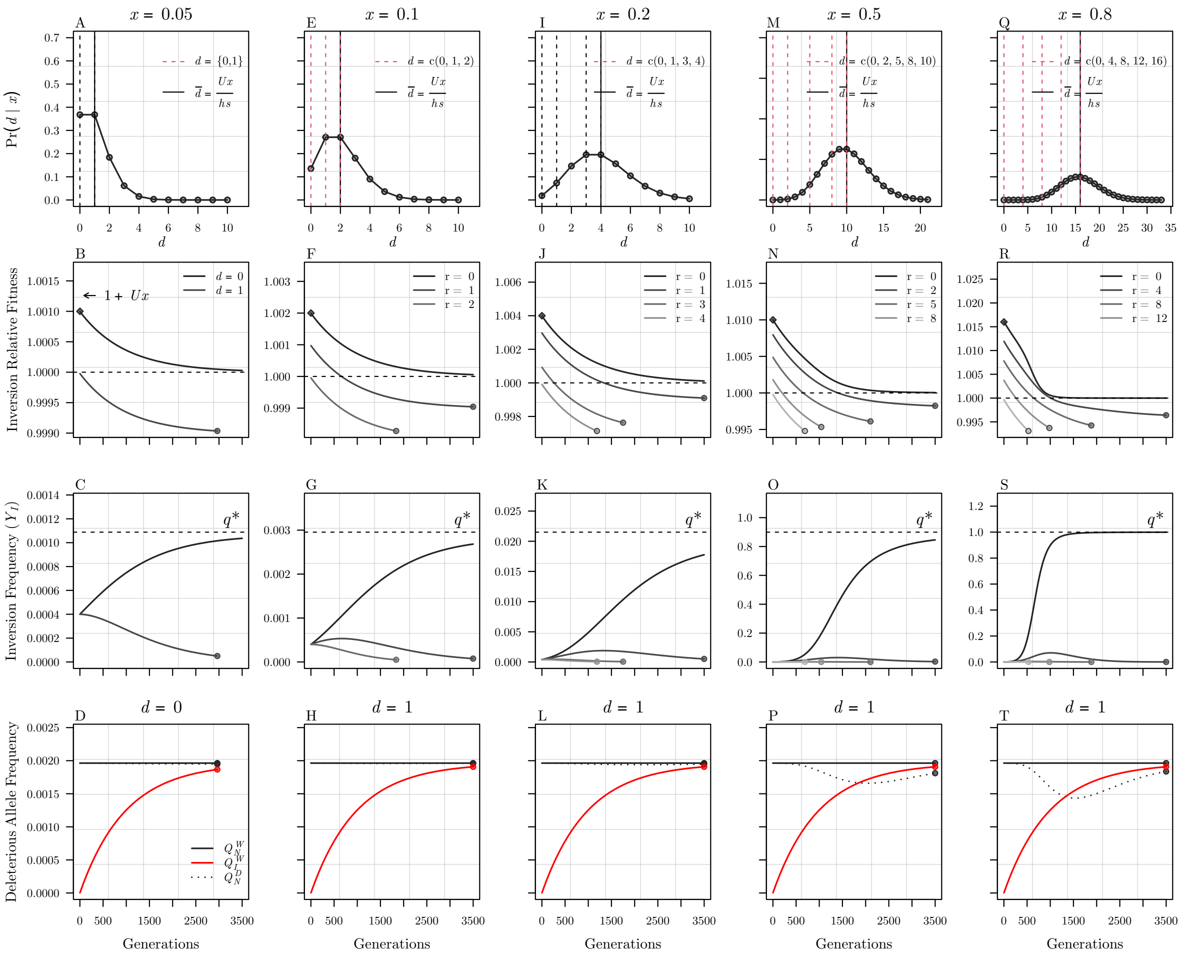

Figure S13: Overview of deterministic fitness and frequency dynamics for initially beneficial **Autosomal inversions** of different sizes initially loaded with different numbers of **strongly recessive** deleterious alleles with a **low mutation rate** ($h = 0.1$ and $U = 0.02$) deleterious alleles. Each column of panels presents results for inversions of lengths $x = 0.05$ (A–D), 0.1 (E–H), 0.2 (I–L), 0.5 (M–P), and 0.8 (Q–T). The first row of panels (A,E,I,M,Q) shows the probability that an inversion of length x captures d deleterious alleles (points and black lines), with benchmarks (vertical red dashed lines) showing the values of d being illustrated in the corresponding column of panels. Values of d were chosen to (more or less) evenly cover the lower half of the distribution of $\Pr(d|x)$. As in Fig. 1 of the main text, the lower three rows of panels illustrate changes in inversion relative fitness (2nd row), inversion frequency (3rd row), and deleterious allele frequencies at *wt* loci on the inversion ($q^W_{Y_I}$; red line), and both *wt* and *del* loci on X chromosomes in ovules/eggs ($q^W_{X_f}$ and $q^D_{X_f}$; black solid and dashed lines respectively) (4th row). Results were generated using the following parameter values: $h = 0.1$, $s = 0.01$, $U = 0.02$, $n_{tot} = 10^4$.

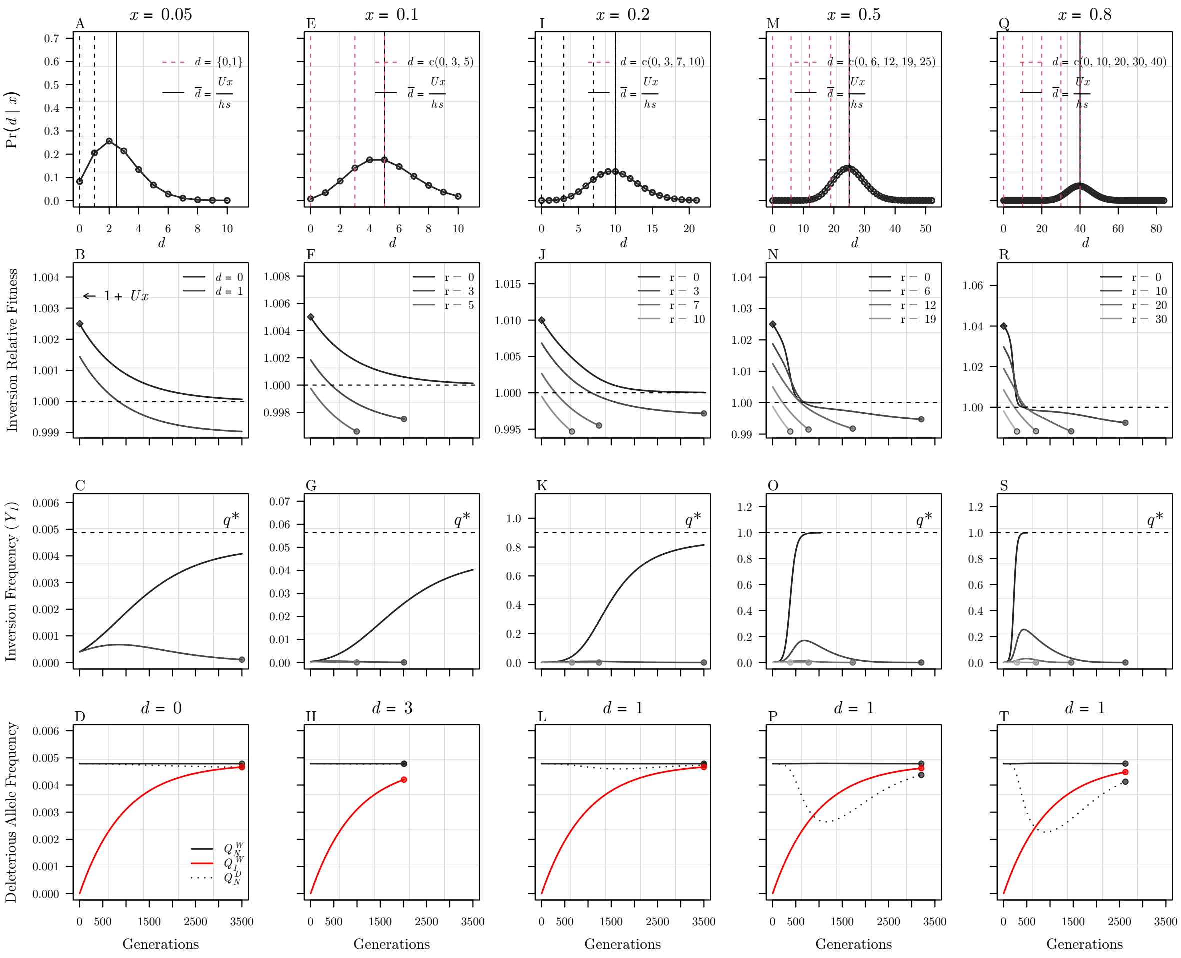

Figure S14: Overview of deterministic fitness and frequency dynamics for initially beneficial **Autosomal inversions** of different sizes initially loaded with different numbers of **strongly recessive** deleterious alleles with an **intermediate mutation rate** ($h = 0.1$ and $U = 0.05$). Each column of panels presents results for inversions of lengths $x = 0.05$ (A–D), 0.1 (E–H), 0.2 (I–L), 0.5 (M–P), and 0.8 (Q–T). The first row of panels (A,E,I,M,Q) shows the probability that an inversion of length x captures d deleterious alleles (points and black lines), with benchmarks (vertical red dashed lines) showing the values of d being illustrated in the corresponding column of panels. Values of d were chosen to (more or less) evenly cover the lower half of the distribution of $\Pr(d|x)$. As in Fig. 1 of the main text, the lower three rows of panels illustrate changes in inversion relative fitness (2nd row), inversion frequency (3rd row), and deleterious allele frequencies at *wt* loci on the inversion ($q^W_{Y_I}$; red line), and both *wt* and *del* loci on X chromosomes in ovules/eggs ($q^W_{X_f}$ and $q^D_{X_f}$; black solid and dashed lines respectively) (4th row). Results were generated using the following parameter values: $h = 0.1$, $s = 0.01$, $U = 0.05$, $n_{tot} = 10^4$.

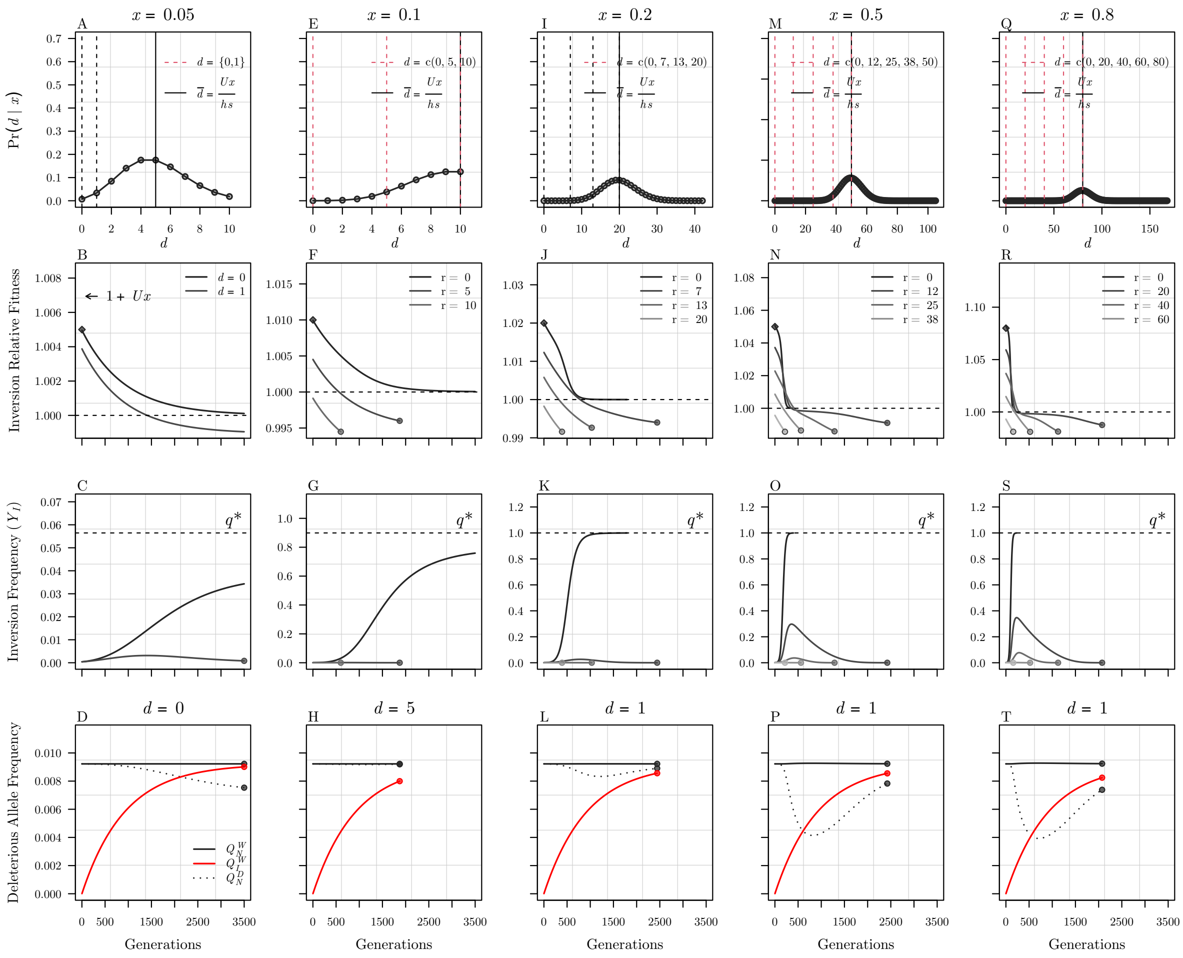

Figure S15: Overview of deterministic fitness and frequency dynamics for initially beneficial **Autosomal inversions** of different sizes initially loaded with different numbers of **strongly recessive** deleterious alleles with a **high mutation rate** ($h = 0.1$ and $U = 0.05$). Each column of panels presents results for inversions of lengths $x = 0.05$ (A–D), 0.1 (E–H), 0.2 (I–L), 0.5 (M–P), and 0.8 (Q–T). The first row of panels (A,E,I,M,Q) shows the probability that an inversion of length x captures d deleterious alleles (points and black lines), with benchmarks (vertical red dashed lines) showing the values of d being illustrated in the corresponding column of panels. Values of d were chosen to (more or less) evenly cover the lower half of the distribution of $\Pr(d|x)$. As in Fig. 1 of the main text, the lower three rows of panels illustrate changes in inversion relative fitness (2nd row), inversion frequency (3rd row), and deleterious allele frequencies at *wt* loci on the inversion ($q^W_{Y_I}$; red line), and both *wt* and *del* loci on X chromosomes in ovules/eggs ($q^W_{X_f}$ and $q^D_{X_f}$; black solid and dashed lines respectively) (4th row). Results were generated using the following parameter values: $h = 0.1$, $s = 0.01$, $U = 0.12$, $n_{tot} = 10^4$.

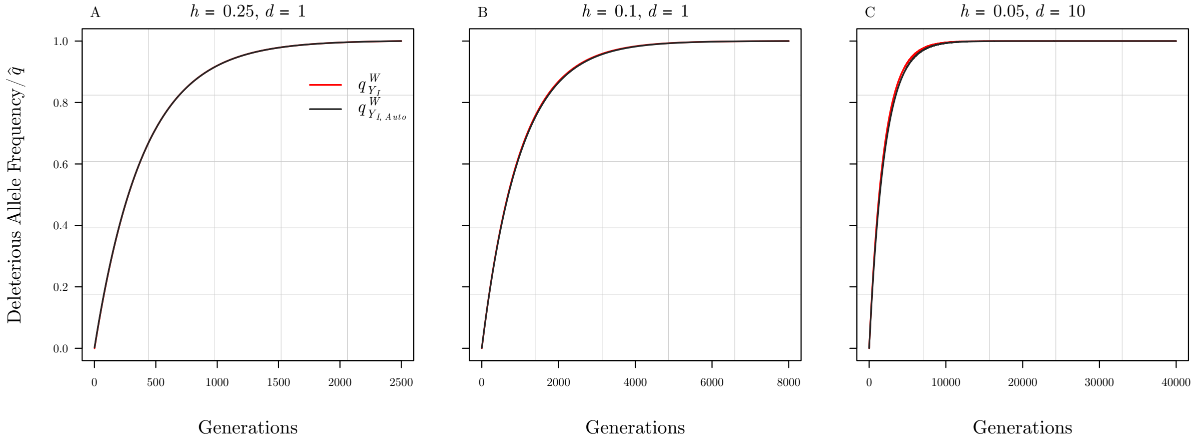

Figure S16: Comparison of deterministic trajectories of deleterious allele frequencies at W loci on descendent copies of autosomal ($q_W^{Y_I,Auto}$; black lines) and SLR-expanding ($q_{Y_I}^W$, red lines) inversions, divided by their corresponding equilibrium frequency ($\hat{q}$). Results are shown for the same parameter values presented in Fig. 1G-I of the main text. When initially beneficial inversions do not deterministically increase in frequency (as in panel A,B above, Fig.1G,H in the main text), there is little to no change in the equilibrium frequency of deleterious alleles on X chromosomes at W loci, and the time-course of deleterious allele accumulation on descendent copies of an SLR-expanding inversion is nearly identical to that of an autosomal inversion. In cases where SLR-expanding inversions do increase in frequency (i.e., when deleterious alleles are strongly recessive as in panel C above, Fig. 1I in the main text), the initially beneficial inversion increases in frequency and the overall frequency of deleterious alleles at W loci on Y chromosomes decreases. This temporary "deficit" of deleterious alleles causes in relaxed purifying selection against deleterious mutations on both X and Y chromsomes, resulting in temporary increases in $q_{X_f}^W$ (see black solid line in Fig. 1I), and slightly faster accumulation of mutations on the inversion.

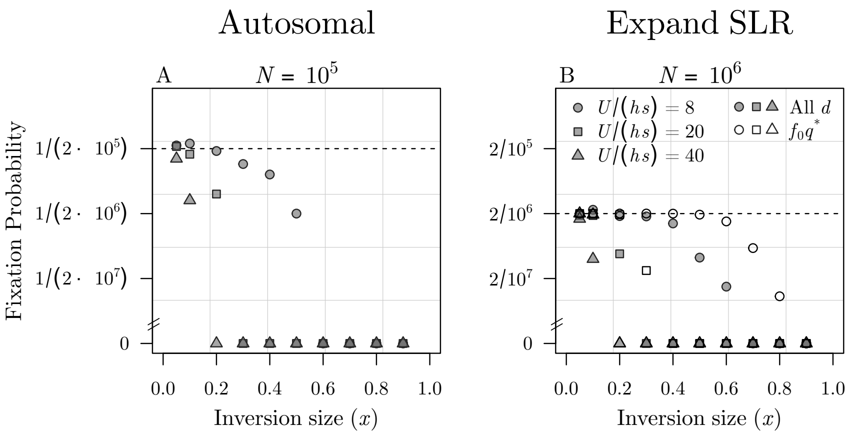

Figure S17: Illustration of how the different WF model predictions begin to diverge **when deleterious mutations are more strongly recessive** ($h = 0.1$). Each figure illustrates inversion fixation probabilities estimated from Wright-Fisher simulations plotted as a function of inversion length for (A) Autosomal and (B) SLR-expanding inversions on Y chromosomes. Point shapes indicate different chromosome-arm wide mutation rates relative to selection (i.e., different values of U), which influences the average deleterious mutation load carried by a standard-arrangement chromosome ($U/(hs)$). Dashed horizontal lines indicate the corresponding expected fixation probability for a neutral variant for the same population size, and hence correspond to values of $1/(2N)$ for autosomal inversions, and $2/N$ for Y-linked inversions. Panel B shows fixation probabilities for all inversions (grey points), and for the analytic approximation given by multiplying the probability that an inversion is initially mutation free by the effective initial frequency (unfilled points; i.e, $\Pr(\text{fix} \mid x) \approx f_0 q^*$). Other parameter values were set to: $h = 0.1$, $s = 0.01$, $n_{tot} = 10^4$, as in figure 2 from the main text.

Appendix F Revisiting the conjecture of Charlesworth & Wall (1999)

As explained in Appendix A, the conjecture of Charlesworth and Wall (1999) is based on the idea that under partial inbreeding, multiple selected loci under deleterious mutation pressure exhibiting associative overdominance will cause similar indirect selection pressures on a structural rearrangement linking them to the SDR as would a single selected locus under heterozygote advantage. Specifically, inbreeding is expected to generate associations between homozygosity and reduced fitness over a chromosome segment having multiple loci with segregating recessive deleterious variation (Charlesworth 1991; Waller 2021). Below, we test this conjecture using a minimal example by extending the simulation model of Charlesworth and Wall (1999) to accommodate three loci: a sex determining locus (SDL), and two selected loci under deleterious mutation pressure (locus **A**, with wild-type allele A and deleterious variant a; and **B**, with corresponding alleles B, and b). In this relatively simple genetic system, the two selected loci should be able to generate the kind of multilocus apparent overdominance proposed by Charlesworth and Wall (1999) when occuring in repulsion phase in double heterozygotes (Charlesworth 1991; Waller 2021).

F.1 Model

The three relevant loci are ordered **SDL** - **A** - **B** on the proto sex chromosomes, and the SDL has two sex-determining "alleles", X and Y, where Y is the dominant male-determining factor. The wild-type alleles at **A** and **B** mutate to deleterious variants at a rate of μ and v per meiosis respectively (with genotypic relative fitness expressions $w_{AA} = 1$, $w_{Aa} = 1 - h_1 s_1$, $w_{aa} = 1 - s_1$, and $w_{BB} = 1$, $w_{Bb} = 1 - h_2 s_2$, $w_{bb} = 1 - s_2$). The recombination rate between the SDL and **A** is denoted q, and that between **A** and **B** is denoted r. The population is assumed to be large, and a fraction, α, of all matings are between full siblings, while the remainder are random. Generations are discrete, and the order of life history events proceeds: (i) mutation, (ii) selection, (iii) meiosis, and (iv) mating.

We track the fate of new inversion mutations that link both selected loci to the SDL and capture a deleterious allele at one of the two selected loci (we arbitrarily assume the new inversion arises on a Y_{Ab} haplotype, yielding an inverted Y_{bA}^{I} haplotype). The conjecture requires that at least one deleterious allele becomes linked to the SDL, and since we also assume no back-mutation, we exclude the inverted Y_{BA}^{I} genotype to reduce the size of the recursion system. Note that in the special case where $q = 1/2$, $r = 0$, $\mu = v = 0$, and w_{ii} terms are given appropriate expressions for heterozygote advantage, the model is mathematically equivalent to the Y-autosome fusion model of Charlesworth and Wall (1999), but with two selected loci (Fig. E1).

F.2 Analysis

This genetic system can be described exactly by a system of 240 recursion equations for the frequencies of matings between all possible pairs of genotypes ($X_{AB}/X_{AB} \times X_{AB}/Y_{AB}$, $X_{AB}/X_{AB} \times X_{AB}/Y_{Ab}$, etc.). The full system of recursions is provided in an accompanying R script in the Online Supplementary Material (Olito et al. 2024). Simulations were carried out in R (R Core Team 2020) for a variety of parameter conditions, but we focus our attention on scenarios of high and low recombination between the three loci, where deleterious mutations are completely or partially recessive ($h_1 = h_2 = \{0.0, 0.1\}$; $s_i = 0.01$). Under these conditions, mild deleterious mutations that are linked in repulsion (haplotypes involving Ab or aB allele pairings) should reduce the fitness of both segregating homozygotes, generating associative-overdominance (also called mutational heterosis) favoring heterozygosity at both loci (Ohta 1971; Waller 2021).

Following Charlesworth and Wall (1999), we introduced a rare inversion haplotype at an initial frequency of 0.001 into a population initially at mutation-selection balance, in males of genotype X_{AB}/Y_{bA}^{I}. Matings involving this

genotype were assumed to be random in the first generation. We then iterated the system of recursions forward in time until the inversion either went extinct or reached a frequency of 0.01. We then calculated the asymptotic rate of increase in log-frequency of the inversion to estimate the overall selection coefficient for the inversion when rare (s_I).

F.3 Simulation results

As a first check that the 3-locus model was correct, we began by reproducing the main result for Y-linked neo-sex chromosome fusions/translocations from Charlesworth and Wall (1999). As noted above (see Appendix A), under the assumption that the selected loci initially recombine freely with the SDL prior to the inversion mutation, a model of an SLR-expanding inversion is equivalent to the models of Charlesworth and Wall (1999) for neo-sex chromosome formation. We ran simulations for the same selection and dominance coefficients they used for heterozygote advantage at the **A** locus, and set the relative fitness of all genotypes at the **B** locus equal to 1 (i.e., the **B** locus is neutral). The simulations faithfully reproduced the main results of increasing invasion fitness with higher rates of partial full-sib mating (Fig. E1).

To explore the effect of linkage between the SDL and selected loci (**A** and **B**) on the conjecture of Charlesworth and Wall (1999), we simulated the invasion of rare SDL-expanding inversions under four different linkage scenarios corresponding to a factorial cross between high and low levels of recombination between the SDL and **A** locus and between the **A** and **B** locus ($q = \{0.5, 0.001\} \times r = \{0.5, 0.001\}$). Under the conjecture, linkage disequilibrium between the selected loci due to inbreeding and/or genetic linkage should promote associative overdominance, resulting in the pair of selected loci behaving similarly to a single locus under heterozygote advantage, as modeled by (Charlesworth and Wall 1999). In this case, an inversion linking a deleterious allele at one locus and a wild-type allele at the other is hypothesized to be increasingly favoured in more inbred populations. However, our simulation results indicate that such an inversion is never selectively favoured under any of the linkage or dominance scenarios (Fig. E2; all selection coefficients for the inveresion are negative). In the most permissive scenario, when deleterious mutations are completely recessive ($h_i = 0$), an SLR-expanding inversion becomes nearly neutral in highly inbred populations.

Taken in the broader context of our other model results presented in the main text, these supplementary simulation results suggest that the conjecture of Charlesworth and Wall (1999) does not work for the following reason: despite potentially strong linkage disequilibrium between selected loci due to both inbreeding and genetic linkage – which should favour associative overdominance at the selected loci – recurrent deleterious mutations ensure that lowfitness homozygous genotypes are continually produced at the locus where the inversion captures a deleterious allele, much as they did in our models of randomly-mating populations. It therefore appears that additional conditions are necessary for this mechanism to favour the invasion of chromosomal inversions (or fusions or translocations between the Y chromosome and an autosome). For example, the models of de Waal Malefijt and Charlesworth (1979), in which autosomal translocation polymorphisms could be maintained under mutational heterosis, involved completely selfing hermaphrodite mating systems in which new deleterious mutations were quickly purged.

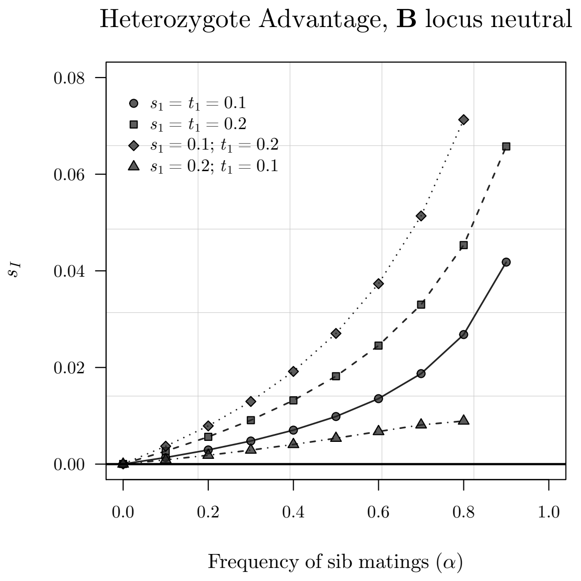

Figure E1: Reproduction of Fig. 1A from Charlesworth and Wall (1999) using the 3-locus model of an SLR-expanding inversion. The figure shows the asymptotic rate of increase in log-frequency of the inversion when rare, which approximates the overall selection coefficient for the inversion, as a function of the frequency of full-sib matings (α). Results are shown for several forms of heterozygote advantage: $s_1 = t_1 = 0.1$; $s_1 = t_1 = 0.2$; $s_1 = 0.1, t_1 = 0.2$; $s_1 = 0.2, t_1 = 0.1$. Cases where there is no polymorphism at equilibrium prior to the inverseion are not shown.

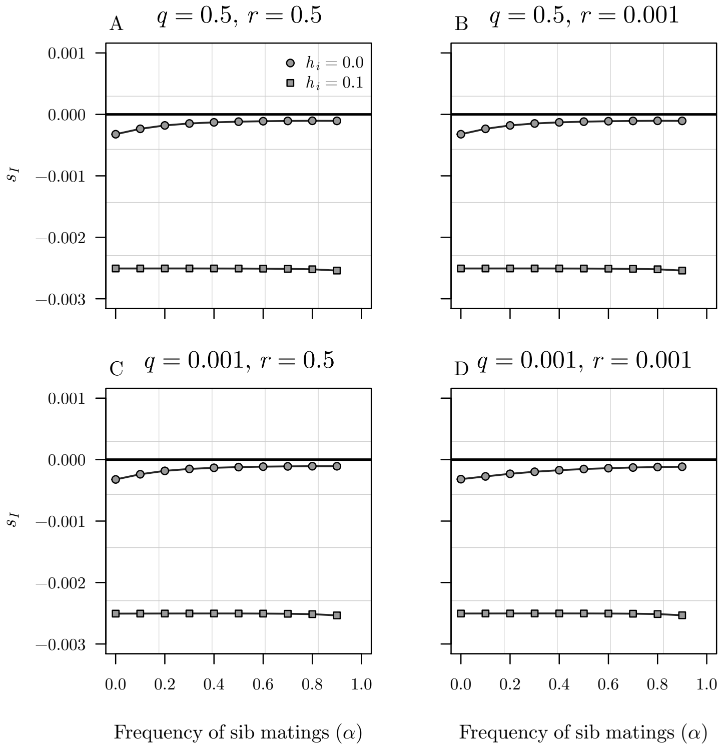

Figure E2: Effect of linkage between the SDL and selected loci (**A** and **B**) on the invasion fitness of an SLR-expanding inversion capturing deleterious alleles in repulsion phase in the 3-locus model. The figure shows the asymptotic rate of increase in log-frequency of the inversion when rare, which approximates the overall selection coefficient for the inversion, as a function of the frequency of full-sib matings (α). Results are shown for four different linkage scenarios corresponding to a factorial cross between high and low levels of recombination between the SDL and **A** locus and between the **A** and **B** locus ($q = \{0.5, 0.001\} \times r = \{0.5, 0.001\}$) and two different dominance scenarios ($h_i = \{0.0, 0.1\}$) for recessive deleterious mutations with a selection coefficient of $s_i = 0.1$ (where $i \in \mathbf{A}, \mathbf{B}$).

Appendix G Revisiting the verbal hypothesis of Branco et al. (2018): Inbreeding and Linkage

As highlighted in Appendix A, the verbal 'sheltering' model proposed by Branco et al. (2017) suggests that prior linkage disequilibrium caused by a combination of inbreeding and partial linkage to mating-type or sex-determining genes makes selection against recessive deleterious alleles less efficient, thereby allowing deleterious alleles to increase in frequency at the margin of the non-recombining region. Rare recombination events are then suggested to generate individuals homozygous for (partially) recessive or deleterious alleles, which are selected against. Below, we develop a simple deterministic 2-locus model to study whether an inversion (or other large-effect recombination modifier) linking a deleterious allele at a single selected locus to the dominant male-determining factor on the Y chromosome can invade a diploid population with X-Y genetic sex determination. Although this scenario is partially addressed by the previous 3-locus simulation model, the simpler 2-locus scenario is permissive of an analytic solution that offers additional insight.

G.1 Model

Consider a large population (i.e., genetic drift is negligible) of diploid, sexually reproducing individuals with discrete generations, in which sex is determined genetically by a dominant male-determining factor (i.e., a male heterogametic X-Y system). Our results are equally applicable to female heterogametic Z-W systems if male- and female-specific parameters are reversed. The population exhibits variation in the rate of inbreeding such that a fraction, α, of the population has an inbreeding coefficient of F, while the remaining fraction $(1-\alpha)$ mates randomly. The gene(s) determining sex reside within a small non-recombining SLR, but recombination still occurs elsewhere along the chromosome at a rate r per meiosis. As in our other models, we assume that genes located outside the SLR have functional homologs on both X and Y chromosomes. Generations are assumed to be discrete, and the order of life history events is: (i) mating, (ii) mutation, (iii) selection, and (iv) meiosis.

We model the evolution of a large-effect recombination modifier (for simplicity we refer to the modifier as a chromosomal inversion) arising on a Y chromosome that (i) expands the SLR to include a single selected locus (the "load locus"); (ii) completely suppresses recombination between inverted and non-inverted karyotypes (in reality genetic exchange may rarely occur via double crossovers or gene conversion; Krimbas and Powell 1992; Korunes and Noor 2019); and (iii) has no direct fitness effects (i.e., has no breakpoint effects and causes no meiotic dysfunction). The wild-type allele (A) at the load locus mutates to a deleterious variant (a) at a rate μ per meiosis (with genotypic relative fitness expressions $w_{AA}=1$, $w_{Aa}=1-hs$, $w_{aa}=1-s$), and is assumed to be under mutation-selection balance prior to the inversion mutation. Fixation of the inversion would lead to expansion of the non-recombining SLR, and fixation of the deleterious a allele among Y chromosomes.

We assume the timescale for loss or fixation of the inversion is sufficiently short that dosage compensation is unlikely to evolve in the chromosomal region it spans. The critical question is whether there is any biologically plausible scenario in which a rare inversion capturing the deleterious variant can invade and fix among Y chromosomes. Below, we provide a brief analysis of this scenario under arbitrary levels of inbreeding and ancestral linkage between the SLR and load locus. A full derivation of the recursions and analysis of this model is provided in the accompanying Mathematica notebook file (.nb).

G.2 Analysis

The evolutionary dynamics of this genetic system can be represented by a system of four haplotype recursions describing the frequency changes of the deleterious a allele at the load locus among the four relevant chromosome classes: X chromosomes among ovules/eggs (X_f), X chromosomes among pollen/sperm (X_m), non-inverted Y chromosomes (Y), and inverted Y chromosomes (Y^I). Under weak selection ($0 < s \ll 1$), the expected change in frequency of the inversion due to selection is expected to be slow relative to that due to inbreeding (Caballero and Hill 1992; Jordan and Connallon 2014; Olito 2017). In this case, it is reasonable to use a separation of timescales to approximate the per-generation change in haplotype frequencies (Otto and Day 2007). Specifically, we calculate the expected genotypic frequencies due to inbreeding in the absence of selection, and then substitute these quasi-equilibrium (QE) frequencies into the haplotype recursions before calculating the per-generation frequency change due to selection and mutation.

To identify parameter conditions under which the rare inversion haplotype is expected to invade, we evaluated the stability of the system of recursions for populations initially at mutation-selection balance (i.e., where $X_f = \hat{X}_f$, $X_m = \hat{X}_m$, $Y = \hat{Y}$, and $Y^I = 0$). Under these assumptions, the overall selection coefficient for the inversion can be approximated by subtracting one from the eigenvalue of the Jacobian matrix associated with the change in frequency of inversion haplotypes ($s_I = \lambda_I - 1$). We are interested in identifying the parameter conditions under which the inversion is selectively favored (where $s_I > 0$). The conditions for the spread of the inversion will be most permissive when deleterious mutations are completely recessive ($h = 0$). In this case, the approximate selection coefficient for the inversion is

$$s_I = \frac{\alpha F(1-X_f) + s(1-Y)\big(\alpha F + 2X_f(1-\alpha F)(1-\mu) + \mu(1-\alpha F)\big)}{s\Big(X_f\Big(\alpha F + 2Y(1-\alpha F)\big(1-\mu+\mu(1-\alpha F)\big) + Y\big(\mu + \alpha F(1-\mu)\big)\Big) - 2\Big)} \tag{F1}$$

where F is Wright's inbreeding coefficient, and X_f and Y are the equilibrium frequencies of the deleterious allele on X chromosomes in ovules/eggs and Y chromosomes prior to the inversion mutation. Evaluation of Eq(F1) reveals that it is negative for all biologically meaningful parameter space (i.e., for $0 \leq \alpha, F, s, \mu, X_f, Y \leq 1$), indicating that no amount of inbreeding or prior linkage between the sex-determining and load loci can generate sufficient linkage disequilibrium to favor inversion establishment.

References

Branco, S., H. Badouin, R. C. Rodríguez de la Vega, J. Gouzy, F. Carbentier, G. Aguileta, S. Siguenza, J.-T. Brandenburg, M. A. Coelho, M. E. Hood, and T. Giraud, 2017. Sex differences in fitness and selection for centric fusions between sex-chromosomes and autosomes. PNAS 114:7067–7072.

Caballero, A. and W. G. Hill, 1992. Effects of partial inbreeding on fixation rates and variation of mutant genes. Genetics 131:493–507.

Charlesworth, B. and J. D. Wall, 1999. Inbreeding, heterozygote advantage and the evolution of neo-x and neo-y sex chromosomes. Proc. Roy Soc. B 266:51–56.

Charlesworth, D., 1991. The apparent selection on neutral marker loci in partially inbreeding populations. Genet. Res. Camb. 57:159–175.

Charlesworth, D. and B. Charlesworth, 2010. Elements of evolutionary genetics. Roberts and Company Publishers, Colorado, USA.

Connallon, T. and C. Olito, 2020. Natural selection and the distribution of chromosomal inversion lengths. Mol. Ecol. .

Ironside, J. E., 2010. No amicable divorce? challenging the notion that sexual antagonism drives sex chromosome evolution. Bioessays 32:718–726.

Jay, P., E. Tezenas, A. Véber, and T. Giraud, 2022. Sheltering of deleterious mutations explains the stepwise extension of recombination suppression on sex chromosomes and other supergenes. PLoS Biology 20:e3001698. Https://doi.org/10.1371/journal.pbio.3001698.

Jordan, C. Y. and T. Connallon, 2014. Sexually antagonistic polymorphism in simultaneous hermaphrodites. Evolution 68:3555–3569.

Korunes, K. L. and M. A. F. Noor, 2019. Pervasive gene conversion in chromosomal inversion heterozygotes. Molecular Ecology 28:1302–1315.

Krimbas, C. B. and J. R. Powell (eds.) 1992. *Drosophila* inversion polymorphism. CRC Press, Florida, USA.

Lenormand, T. and D. Roze, 2022. Y recombination arrest and degeneration in the absence of sexual dimorphism. Science 375:663–666.

———, 2024. Can mechanistic constraints on recombination reestablishment explain the long-term maintenance of degenerate sex chromosomes? Peer Community Journal 4:e17. Doi: 10.24072/pcjournal.373.

Nei, M., K.-I. Kojima, and H. E. Schaffer, 1967. Frequency changes of new inversions in populations under mutation-selection equilibria. Genetics 57:741–750.

Ohta, T., 1971. Associative overdominance caused by linked detrimental mutations. Genet. Res. Camb. 18:277–286.

Olito, C., 2017. Consequences of genetic linkage for the maintenance of sexually antagonistic polymorphism in hermaphrodites. Evolution 71:458–464.

Olito, C. and B. Charlesworth, 2023. Do deleterious mutations promote the evolution of recombination suppression between x and y chromosomes? bioR χiv Pp. 1–9.

Olito, C., B. Hansson, S. Ponnikas, and J. K. Abbott, 2024. Consequences of partially recessive deleterious genetic variation for the evolution of inversions suppressing recombination between sex chromosomes: Computer code. Zenodo Digital Repository doi: 10.5281/zenodo.6361985.

Otto, S. and T. Day, 2007. A biologist's guide to mathematical modeling in ecology and evolution. Princeton University Press, New Jersey, USA.

Ponnikas, S., H. Sigeman, J. K. Abbott, and B. Hansson, 2018. Why do sex chromosomes stop recombining? Trends in Genetics 34:492–503.

R Core Team, 2020. R: A Language and Environment for Statistical Computing. R Foundation for Statistical Computing, Vienna, Austria. URL `https://www.R-project.org/`.

de Waal Malefijt, M. and B. Charlesworth, 1979. A model for the evolution of translocation heterozygosity. Heredity 43:315–331.

Waller, D. M., 2021. Addressing darwin's dilemma: can pseudo-overdominance explain persistent inbreeding depression and load? Evolution 75:779–793.
